# Expanding the Clinical and Genetic Spectra of Primary Immunodeficiency-Related Disorders With Clinical Exome Sequencing: Expected and Unexpected Findings

**DOI:** 10.3389/fimmu.2019.02325

**Published:** 2019-10-01

**Authors:** Francesc Rudilla, Clara Franco-Jarava, Mónica Martínez-Gallo, Marina Garcia-Prat, Andrea Martín-Nalda, Jacques Rivière, Aina Aguiló-Cucurull, Laura Mongay, Francisco Vidal, Xavier Solanich, Iñaki Irastorza, Juan Luis Santos-Pérez, Jesús Tercedor Sánchez, Ivon Cuscó, Clara Serra, Noelia Baz-Redón, Mónica Fernández-Cancio, Carmen Carreras, José Manuel Vagace, Vicenç Garcia-Patos, Ricardo Pujol-Borrell, Pere Soler-Palacín, Roger Colobran

**Affiliations:** ^1^Immunogenetics and Histocompatibility Laboratory, Banc de Sang i Teixits, Barcelona, Spain; ^2^Transfusional Medicine Group, Vall d'Hebron Research Institute, Autonomous University of Barcelona, Barcelona, Spain; ^3^Immunology Division, Department of Cell Biology, Physiology and Immunology, Vall d'Hebron Research Institute, Hospital Universitari Vall d'Hebron, Autonomous University of Barcelona, Barcelona, Spain; ^4^Jeffrey Model Foundation Excellence Center, Barcelona, Spain; ^5^Pediatric Infectious Diseases and Immunodeficiencies Unit (UPIIP), Hospital Universitari Vall d'Hebron, Vall d'Hebron Research Institute, Autonomous University of Barcelona, Barcelona, Spain; ^6^CIBER on Cardiovascular Diseases (CIBERCV), Instituto de Salud Carlos III (ISCIII), Valencia, Spain; ^7^Adult Immunodeficiencies Unit (UFIPA), Internal Medicine Department, Institut d'Investigació Biomèdica de Bellvitge, Hospital Universitari de Bellvitge, L'Hospitalet de Llobregat, Barcelona, Spain; ^8^Pediatric Gastroenterology, Cruces University Hospital, Basque Country University, Bilbao, Spain; ^9^Immunodeficiencies and Infectious Disease Unit, Universitary Hospital Virgen de las Nieves, Granada, Spain; ^10^Unidad de Dermatología Pediátrica y Anomalías Vasculares, Servicio de Dermatología, Instituto de Investigación Biosanitaria IBS, Hospital Universitario Virgen de las Nieves, Granada, Spain; ^11^Genetics Department, Hospital Universitari Vall d'Hebron, Barcelona, Spain; ^12^Growth and Development Group, Vall d'Hebron Research Institute, Hospital Universitari Vall d'Hebron, Autonomous University of Barcelona, Barcelona, Spain; ^13^CIBER Rare Diseases (CIBERER), Instituto de Salud Carlos III (ISCIII), Madrid, Spain; ^14^Pediatric Hematology and Immunodeficiencies Unit, Hospital Universitario y Politécnico La Fe, Valencia, Spain; ^15^Hematology Department, Complejo Hospitalario Universitario de Badajoz, Badajoz, Spain; ^16^Dermatology Department, Hospital Universitari Vall d'Hebron, Barcelona, Spain

**Keywords:** primary immunodeficiencies, next generation sequencing, clinical exome sequencing, TruSight one sequencing panel, mutations, genetic variants

## Abstract

Primary immunodeficiencies (PIDs) refer to a clinically, immunologically, and genetically heterogeneous group of over 350 disorders affecting development or function of the immune system. The increasing use of next-generation sequencing (NGS) technology has greatly facilitated identification of genetic defects in PID patients in daily clinical practice. Several NGS approaches are available, from the unbiased whole exome sequencing (WES) to specific gene panels. Here, we report on a 3-year experience with clinical exome sequencing (CES) for genetic diagnosis of PIDs. We used the TruSight One sequencing panel, which includes 4,813 disease-associated genes, in 61 unrelated patients (pediatric and adults). The analysis was done in 2 steps: first, we focused on a virtual PID panel and then, we expanded the analysis to the remaining genes. A molecular diagnosis was achieved in 19 (31%) patients: 12 (20%) with mutations in genes included in the virtual PID panel and 7 (11%) with mutations in other genes. These latter cases provided interesting and somewhat unexpected findings that expand the clinical and genetic spectra of PID-related disorders, and are useful to consider in the differential diagnosis. We also discuss 5 patients (8%) with incomplete genotypes or variants of uncertain significance. Finally, we address the limitations of CES exemplified by 7 patients (11%) with negative results on CES who were later diagnosed by other approaches (more specific PID panels, WES, and comparative genomic hybridization array). In summary, the genetic diagnosis rate using CES was 31% (including a description of 12 novel mutations), which rose to 42% after including diagnoses achieved by later use of other techniques. The description of patients with mutations in genes not included in the PID classification illustrates the heterogeneity and complexity of PID-related disorders.

## Introduction

Primary immunodeficiencies (PIDs) are a phenotypically and genetically heterogeneous group of inborn errors of immunity leading to a predisposition to infections, autoimmune, or autoinflammatory diseases, lymphoproliferation, and malignancies. Typically considered as rare diseases (PID prevalence ranges from 1:10,000 to 1:100,000 population) recent studies indicate that they are more common than was formerly believed (1). The diagnostic workup for PIDs has advanced from clinical evaluation with a detailed personal and family history to a more recent series of complex laboratory assays, including extensive flow cytometry studies, cell culture, or western blotting. As most described PIDs have a monogenic cause, molecular genetic testing is usually the key to providing a definite diagnosis (2). A positive genetic diagnosis can direct the patient toward suitable prevention, monitoring, and treatment options. In addition, the genetic results can help patients make choices regarding their future, especially in terms of genetic counseling about having children. In the case of inherited mutations, testing of relatives is also important to expand medical care, and genetic counseling to all affected family members and carriers. However, achieving a definite genetic diagnosis in a suspected case of PID can be a complex and laborious process that sometimes fails to yield positive results.

Identification of the numerous monogenic defects underlying PIDs has continuously increased over time. Evidence of this is seen in the current IUIS report, which, as of February 2017, included 354 distinct disorders with 344 different gene defects listed (3). Remarkably, in the previous 2 years (2015–2016), 85 new causal genes were identified (4). This striking increase in the number of disorders recognized in the last years is undoubtedly driven by the increasingly more extensive use of next-generation sequencing (NGS) technology. NGS has greatly accelerated the discovery of novel disease-causing genes and facilitated the genetic diagnosis of patients with monogenic inborn errors of immunity (5). Several NGS approaches, ranging from the unbiased whole exome sequencing (WES) and whole genome sequencing (WGS) to the more restricted targeted gene panel sequencing have been successfully used for the diagnosis of PIDs (6). The efficacy of NGS in terms of the percentage of positive diagnoses is difficult to establish, as it is highly dependent of the type of PID suspected, but in general it does not reach 50% in the various cohorts reported (7–11). Several factors may explain this relatively low percentage of success, including the existence of complex models of inheritance (e.g., oligogenic, polygenic, epigenetic) which are just beginning to be addressed by the scientific community (12, 13). Moreover, the patients included in these studies often show highly heterogeneous PID symptoms and many are complex cases that remain genetically unresolved after using traditional sequential Sanger sequencing procedures. The other side of the coin of investigating challenging patients is that novel PID-associated genes have been discovered and unusual phenotypes caused by mutations in genes known to cause PID have been identified (6). Hence, the use of NGS has expanded our knowledge of the complex clinical phenotypes associated with PID and has refined phenotype-genotype correlations in many cases. Certain studies with more defined cohorts in terms of PID suspicion have obtained higher percentages of positive diagnoses. An example is the recent study by Yu et al. focused on molecular analysis of infants with abnormal T-cell receptor excision circles (TRECs), positive newborn screening results, or a positive family history of PID. Using a customized NGS-based multigene-targeted panel for SCID and other severe PIDs, these authors identified disease-causing mutations in 14 of 20 patients (70%) (14).

Clinical exome sequencing (CES) represents an intermediate step between analysis by specific gene panels and WES. WES relies on investigating all protein-coding regions of the genome (i.e., exons + flanking regions), and because most known disease-causing mutations occur in exons, WES is considered an effective method to identify them. However, while hundreds of genetic conditions have been fully characterized at the clinical and molecular level, most human genes are not actually related to human diseases. CES focuses on known disease-associated genes and provides a rapid and cost-effective sequencing, analysis, and interpretation of the results.

Here, we report our experience using CES for the genetic diagnosis of PIDs. The study included 61 children and adults, and the molecular diagnosis was reached in 19 of them (31%). The results are presented after dividing patients into those with mutations in PID-causing genes (based on the IUIS classification) and those with mutations in genes not included in the PID classification. These latter cases have yielded interesting and somewhat unexpected findings that expand the clinical and genetic spectra of PID and PID-related disorders. We also discuss the limitations of CES, exemplified by patients with negatives results on this test, but later diagnosed by other methods, such as more specific PID panels, WES, and array comparative genomic hybridization (aCGH).

## Materials and Methods

### Patients and Samples

This study included 61 unrelated patients attended during a 3-year period (2015–2017). Most patients were attended at Hospital Universitari Vall d'Hebron (HUVH) Barcelona (Spain), but as HUVH is a reference center for the diagnosis of PID in Spain, samples from other regions of the country were also included. All patients underwent a clinical history and laboratory analyses (including immunophenotyping and functional tests), which pointed to suspected PID. Clinical data were obtained from the patient's medical chart. In some patients, direct sequencing (Sanger method) of suspected genes had been performed previously with negative results. DNA was isolated using different methods, but ultimately, all samples were precipitated and resuspended with pure water to homogenize all DNAs.

Written informed consent for the studies reported here and for the publication was obtained from the patients or their legal representatives, according to the procedures of the Ethics Review Board of Hospital Universitari Vall d'Hebron [code: PR(AG)69/2016].

### Clinical Exome Sequencing and Data Analysis

CES was carried out using the TruSight One (TSO) Sequencing Panel (Illumina, San Diego, CA, USA) according to the manufacturer's instructions. The panel covers 4,813 disease-associated genes. Targeted exonic regions underwent paired-end sequencing on an Illumina platform using a MiSeq or NextSeq 500 sequencing system (NextSeq High Output Kit, 300 cycles). The MSR:Enrichment v2.4.60.8 and BWA Enrichment v2.1.1-v2.1.2 packages (BaseSpace, Illumina) were used to align the resulting sequence reads to a reference genome (GRCh37, hg19) (Burrows-Wheeler Aligner, BWA) and to carry out variant calling (Genome Analysis Toolkit, GATK) (15, 16). The Isaac Enrichment v1.0 and Enrichment v2.1.0–v3.0.0 tools were also used for alignment and variant calling (BaseSpace, Illumina). The sequencing quality parameters obtained from the MiSeq sequencing platform are shown in Supplementary Table 1. Considering the average value of a set of 20 runs with 3 samples in each sequencing run, more than 23 million mapped reads per run were obtained in a cluster density of 1,183 K/mm^2^, with the cluster PF being 88.6%. In total, 89.7% of bases showed a desirable quality score of ≥Q30. Two sequencing runs of 36 samples each were performed using the NexSeq 500 platform (Supplementary Table 1). The cluster density was 151 K/mm^2^, the cluster PF was 86.5%, and there were 93 million targeted reads. The percentage of bases with ≥Q30 were within specifications in both runs, with a mean of 80.5%.

The data analysis of relevant disease variants was carried out with the Illumina Variant Studio software v3.0 and BaseSpaceVariant Interpreter Beta (Illumina). All variants were obtained using the quality parameters established by the filter pass in the above-mentioned software. Variants considered in this analysis were single nucleotide changes and short insertions or deletions. PolyPhen-2 and SIFT were used to predict the pathogenicity of the missense variants detected (17, 18). All pathogenic variants were confirmed by Sanger sequencing.

## Results and Discussion

### Overall Sequencing Data and Results Summary

In this study, we report the CES results in 61 unrelated patients with a clinically suspected PID. DNA samples were sequenced using the TSO sequencing panel, which covers the exons and flanking regions of 4,813 disease-associated genes. The mean target coverage depth was 81 ± 28X, with a mean 20X target coverage of 89 ± 4% (Supplementary Table 2). Biological data analysis involved 2 steps: First, we analyzed the variants in a virtual PID panel of 260 genes that contained the TSO genes included in the current IUIS classification (Supplementary Table 3) (3), and then, we extended the analysis to the full TSO gene panel in patients with negative results in the virtual PID panel.

Sixty-one patients were included in the study, 36 males (59%) and 25 females (41%), with a mean age of 11 years at enrollment (range, 0–57 years); 82% were pediatric patients (50/61). Eighteen percent of patients were non-Caucasian, and 23% had a family history of consanguinity. The patients' epidemiologic data are summarized in Table 1. A genetic cause explaining the patient's phenotype was found in 31% of patients (19/61), a percentage comparable to the rates reported in other NGS-based studies (8–10, 19–21). Among these positive genetic results, 12 were associated with genes included in the latest IUIS classification (3), and 7 involved genes that are not typically associated with classical PID phenotypes. Additionally, 5 patients showed variants of uncertain significance (VUS) or incomplete genotypes in genes related to their clinical phenotype. Of the 39 patients without a positive genetic diagnosis by CES, 7 (11%) were successfully diagnosed later using other approaches. CES had failed for the following reasons: (1) the genes were not included in the CES panel, (2) large genomic rearrangements were not successfully detected by CES, and (3) mutations occurred in non-coding regions, which are beyond the scope of CES. In the following sections, these results are described in detail and discussed. The clinical and laboratory data of the patients included in the next sections are summarized in Tables 2, 3. The clinical and laboratory data of all patients (*n* = 61) included in the study are summarized in Supplementary Tables 4, 5.

**Table 1:** Epidemiology and results summary.

**Total number of patients studied**	**61**
Female	25 (41%)
Male	36 (59%)
Mean age in years (range)	11 (0–57)
Pediatric (<18 years)	50 (82%)
Adult (>18 years)	11 (18%)
**1. Patients with a genetic defect identified by CES**	19 (31%)
Pediatric (% success among pediatric patients)	14 (28%)
Adult (% success among adult patients)	5 (45%)
**2. Incomplete genotypes**/**VUS**	5 (8%)
**3. Patients with a genetic defect identified after CES**	7 (11%)
Because gene was not included in TSO	4
Other reasons	3
**4. Patients without relevant genetic findings**	30 (49%)

*CES, clinical exome sequencing; TSO, TruSight One Sequencing Panel; VUS, variant of uncertain significance*.

**Table 2:** Main clinical features of patients with relevant genetic findings.

**ID**	**Sex**	**Age at study (y)**	**Gene**	**Family history**	**Consanguinity**	**Age of onset (y)**	**Syndromic features**	**Infections**	**Autoimmunity**	**Lymphoproliferation**	**Inflammation**	**Main clinical features, key words**	**Treatment**
**PATIENTS WITH MUTATIONS IN PID GENES (2017 IUIS CLASSIFICATION)**													
P1	M	15	PIK3R1	–	–	7	–	V	–	Malign	Gut	IgA deficiency, immune dysregulation, growth delay, enteropathy, intestinal large bowel lymphoma	IVIG, immunosuppression (rapamycin and rituximab)
P2	F	57	TNFRSF13B	–	–	14	–	B	–	Benign	Gut, lung	CVID, infections, GLILD, granulomas, adenopathies, pancytopenia	IVIG, immunosuppression (rituximab, corticoids, azathioprine), antibiotics
P3	F	8	TNFRSF13B	√	–	3	–	B, V	√	Benign	–	EBV, pancytopenia, hepatosplenomegaly, hypogammaglobulinemia, ALPS-like, CVID-like	IVIG, rapamycin
P4	M	18	IKBKG	√	–	14	–	–	–	–	Gut, joints	Crohn's disease, joint affectation, CGD, NEMO deficiency	Infliximab
P5	F	10	STAT3	–	–	4	√	B, F, Myc	–	–	–	Hyper IgE, atypical mycobacteria	Antifungal prophylaxis, vitamin D, IVIG, antibiotics
P6	M	2	XIAP	–	√	0.8	√	B	–	–	Gut	Enteropathy, bacterial infection, growth delay	IVIG, patient died
P7	M	1	G6PD	–	–	0.3	–	B	–	–	Bone	Osteomyelitis, *Salmonella* spp.	-
P8	M	30	STAT1	–	–	0.0	–	F	√	–	Skin, mucosa	Dermatophytosis, oral candidiasis	Antifungal prophylaxis,
P9	F	4	STAT1	–	–	0.8	–	F	–	–	Joints	CMC, polyarthritis, episcleritis, bronchiectasis	Ruxolitinib, HSCT
P10	M	6	STAT1	√	√	4	–	F	–	–	–	Familial CMC	Antifungal prophylaxis
P11	F	12	PLCG2	–	–	0.8	√	B	–	–	Skin, lungs	Agammaglobulinemia, severe cutaneous inflammation, bronchiectasis, B cell lymphopenia, growth delay	IVIG, antibiotics, corticosteroids, etanercept, anakinra
P12	M	15	ADA	–	–	8	–	B	–	Malign	–	Hodgkin lymphoma, B cell deficiency	Chemotherapy
**PATIENTS WITH MUTATIONS IN GENES NOT INCLUDED IN THE PID CLASSIFICATION**													
P13	M	5	SKIV2L	–	–	0.8	√	B	–	–	Gut	Inflammatory enteropathy, growth delay	Antibiotics, IVIG, adalimumab, immunosuppression (azathioprine, prednisone, rapamycin)
P14	M	0.25	MMACHC	√	–	0.1	–	–	–	–	–	fHLH, XLP	Died before diagnosis
P15	F	44	SLC27A4	–	√	n.a.	–	–	–	–	Skin	Netherton syndrome	-
P16	F	0.3	DSG1	–	–	0.3	–	B	–	–	–	Erythroderma, Netherton syndrome, hyper IgE, eosinophilia	Infliximab, adalimumab, cyclosporine
P17	F	1	DNAI2	√	√	0.1	–	V, F	√	Malign	–	Recurrent bronchitis, biphenotypic leukemia, growth delay	HSCT (due to leukemia)
P18	M	38	SIX6	√	√	n.a.	–	B	–	–	–	CID, Low IgA	IVIG
P19	M	5	RECQL4	–	√	1	√	V, F	–	–	–	CVID, growth delay	IVIG, antibiotic prophylaxis
**PATIENTS WITH INCOMPLETE GENOTYPES/VARIANTS OF UNCERTAIN SIGNIFICATE (VUS)**													
P20	M	11	UNC13D	√	–	8	–	V	–	Malign	Gut	Pancytopenia, hepatosplenomegaly, hemophagocytosis, panniculitic T cell lymphoma	HSCT (due to T cell lymphoma)
P21	M	13	RAG2	√	–	6	√	–	–	Benign	Systemic	Persistent fever, intermittent abdominal pain, granulomatous hepatitis	Immunosuppression (methotrexate + colchicine + corticosteroids)
P22	M	4	PLCG2	√	√	1	–	V	–	–	Skin	Periodic fever, skin rash	-
P23	F	11	TRAF3	–	–	11	–	V	–	–	–	Herpes Zoster, VZV meningoencephalitis	-
P24	M	2	NOD2	–	–	0.6	–	–	–	–	Gut	Early-onset colitis	-
**PATIENTS WITH GENETIC FINDINGS POST-CLINICAL EXOME**													
P25	F	8	LRBA	√	–	0.7	–	B, V, F	√	Malign	Gut	EBV, lymphoproliferation, autoimmunity, infections, dysregulation, enteropathy, autoimmune cytopenia	IVIG, rapamycin, antibiotic prophylaxis, HSCT
P26	F	11	LRBA	–	–	2	–	V	√	Benign	–	ALPS	IVIG, abatacept
P27	F	7	LRBA	–	–	7	–	B	√	Benign	–	Antibody deficiency, autoimmunity, lymphoproliferative syndrome	IVIG, abatacept
P28	F	14	IKZF1	√	–	5	√	B	–	–	–	Agammaglobulinemia, neurological delay	IVIG
P29	M	9	13 Mb del cr.6	√	√	0.0	√	B, V	–	–	–	Neutrophilic dermatosis, oral and genital aphthae, growth delay	Etanercept
P30	M	6	BTK	–	–	4	–	B	–	–	–	Pneumonia, hypogammaglobulinemia, absence of B cells	IVIG
P31	M	25	Gorham-Staut disease	–	–	21	√	B	–	–	Joints	Osteopenia, chylothorax, lymphopenia, *S.Aureus* bacteremia, septic arthritis	-

*n.a., not available; –, no; V, virus; B, bacteria; F, fungi; Myc, mycobacteria; ALPS, autoimmune lymphoproliferative syndrome; CID, combined immunodeficiency; CGD, chronic granulomatous disease; CMC, chronic mucocutaneous candidiasis; CVID, common variable immunodeficiency; EBV, Epstein-Barr virus; fHLH, familial hemophagocytic lymphohistiocytosis; GLILD, granulomatous and lymphocytic interstitial lung disease; HSCT, hematopoietic stem cell transplant; IVIG, intravenous immunoglobulin; VZV, varicella-zoster virus; XLP, X-linked lymphoproliferation. √, The patient presents this feature*.

**Table 3:** Laboratory data of patients with relevant genetic findings.

**ID**	**Sex**	**Age at study (y)**	**Gene**	**Hypogammaglobulinemia**	**Neutropenia**	**Lymphopenia**	**Thrombocytopenia**	**Immunophenotype**	**Functional tests**
**PATIENTS WITH MUTATIONS IN PID GENES (2017 IUIS CLASSIFICATION)**									
P1	M	15	PIK3R1	–	–	–	–	Inverse CD4/CD8. No switched memory B cells	Low proliferation with anti-CD3, altered *in vivo* response to *Haemophilus influenzae*
P2	F	57	TNFRSF13B	√	√	√	√	Low CD4 naïve T cells, low Tregs, low pre-switched and switched B cells	n.a.
P3	F	8	TNFRSF13B	√	√	√	√	Low CD4 naïve T cells, low pre-switched and switched B cells	Normal proliferation, cytotoxicity and degranulation
P4	M	18	IKBKG	–	–	–	–	Increased DN gamma delta T cells (22%)	Normal respiratory burst test and cytotoxicity/degranulation assays
P5	F	10	STAT3	–	–	–	–	Low Tregs, low Th17	Very low proliferation with anti-CD3
P6	M	2	XIAP	–	–	–	–	n.a.	n.a.
P7	M	1	G6PD	–	–	–	–	Inverse CD4/CD8 ratio. Increased effector HLA-DR+ CD8+ T cells, low switched B cells	Very low proliferation with anti-CD3 and ConA, very low IFNγ and IL12 production
P8	M	30	STAT1	–	–	–	–	Myeloid DC >>> plasmacytoid DC	Negative *in vivo* response to Candidin.
P9	F	4	STAT1	–	–	–	–	Low CD4 naïve T cells, low Th17, low pre-switched B cells	Low proliferation with PHA
P10	M	6	STAT1	–	–	–	–	3.4% DN TCR αβ T cells, increased Th1, low Th17, low Tregs	Low IL12 and IFNγ production
P11	F	12	PLCG2	–	–	–	–	T+ B- NK+	Normal proliferation, normal respiratory burst test
P12	M	15	ADA	√	–	–	–	T+ B- NK-	Normal respiratory burst test
**PATIENTS WITH MUTATIONS IN GENES NOT INCLUDED IN THE PID CLASSIFICATION**									
P13	M	5	SKIV2L	√	–	–	–	Normal	Low proliferation with PHA (normal to anti-CD3 and ConA), normal respiratory burst test
P14	M	0.25	MMACHC	–	√	–	√	n.a.	Absent degranulation and cytotoxicity
P15	F	44	SLC27A4	–	–	–	–	Normal	Normal
P16	F	0.3	DSG1	–	–	–	–	Very low effector and memory T cells, low Th2, low Th1/Th17	n.a.
P17	F	1	DNAI2	–	–	–	–	Normal	Low proliferation with PWM and PHA
P18	M	38	SIX6	–	–	–	√	Low switched memory B cells	Low proliferation with anti-CD3
P19	M	5	RECQL4	√	–	√	√	Increased CD4/CD8 ration	Low proliferation with PWM, anti-CD3, and ConA.
**PATIENTS WITH INCOMPLETE GENOTYPES**/**VARIANTS OF UNCERTAIN SIGNIFICANCE (VUS)**									
P20	M	11	UNC13D	–	√	√	–	Normal	Alternate low/normal degranulation and cytotoxicity
P21	M	13	RAG2	–	–	√	√	Low T cells, low NK cells.	Normal proliferation, normal respiratory burst test
P22	M	4	PLCG2	–	√	–	–	Normal	n.a.
P23	F	11	TRAF3	–	–	–	–	Normal	Absent IL-12 production, normal IFNγ production
P24	M	2.0	NOD2	–	–	–	–	Normal	Normal proliferation, normal respiratory burst test
**PATIENTS WITH GENETIC FINDINGS POST-CLINICAL EXOME**									
P25	F	8	LRBA	√	√	√	–	T+ B- NK-	Low/absent degranulation and cytotoxicity
P26	F	11	LRBA	–	–	–	–	4.2% DN TCR αβ T cells, low effector and memory T cells, low Tregs	Low CD69 and CD40L expression
P27	F	7	LRBA	√	–	–	–	Low pre-switched and switched B cells	Low pneumococcal response
P28	F	14	IKZF1	√	–	–	–	T+ B- NK+, low pre-switched and switched B cells	Negative ASLO
P29	M	9	13 Mb del cr.6	–	–	–	–	Normal	Low proliferation with anti-CD3, normal TNFα production in response to LPS, normal degranulation and cytotoxicity
P30	M	6	BTK	√	–	–	–	T+ B- NK+	n.a.
P31	M	25	Gorham-Staut disease	–	√	–	–	Low CD4 T cells, increased NK cells	Severe defect in IL12 production, altered respiratory burst test

*n.a., not available; -, no; ASLO, anti-streptolysin O; ConA, concanavalin A; DC, dendritic cell; DN, double negative; PHA, phytohemagglutinin; PMA, phorbol 12-myristate 13-acetate; PWM, pokeweed mitogen. √, The patient presents this feature*.

### Patients With Mutations in PID Genes (2017 IUIS Classification)

The filtering strategy for PID genes included in the current IUIS classification led to a molecular diagnosis in 12 cases (Table 4). Mean age in this group was 14.8 years (range, 1–57), and the male-to-female ratio was 1.4.

**Table 4:** Relevant genetic findings in patients included in this study.

**Patient**	**Gene**	**Chr**	**OMIM**	**cDNA**	**Protein**	**Consequence**	**Zygosity**	**Inheritance**	**dbSNP ID**	**ExAc MAF**	**PolyPhen2**	**SIFT**	**Previous report of variant**
**MUTATIONS IN PID GENES (2017 IUIS CLASSIFICATION)**													
P1	PIK3R1	5	616005	c.1425+1G>A		Splicing defect	Heterozygous	AD	rs587777709				(22, 23)
P2	TNFRSF13B	17	240500	c.310T>C	p.Cys104Arg	Missense	Heterozygous	AR, AD	rs34557412	0.003212	Deleterious (0)	probably damaging (1)	(24, 25)
P3	TNFRSF13B	17	240500	c.260T>A	p.Ile87Asn	Missense	Heterozygous	AR, AD	rs72553877	0.000461	Deleterious (0)	probably_damaging (0.924)	(26, 27)
P4	IKBKG	X	300291	c.169G>A	p.Glu57Lys	Missense	Hemizygous	X-linked	rs148695964	0.001190	Deleterious (0.01)	probably_damaging (0.995)	(28, 29)
P5	STAT3	17	147060	c.1144C>T	p.Arg382Trp	Missense	Heterozygous	AD	rs113994135		Deleterious (0)	probably_damaging (0.999)	(30, 31)
P6	XIAP	X	300635	c.888_892del	p.Lys299LeufsX9	Frameshift	Hemizygous	X-linked					Novel
P7	G6PD	X	300908	c.934G>C	p.Asp312His	Missense	Hemizygous	X-linked	rs137852318	0.000840	Deleterious (0)	probably_damaging (0.911)	(32)
P8	STAT1	2	614162	c.1060C>G	p.Leu354Val	Missense	Heterozygous	AD			Tolerated (0.19)	benign (0.363)	Novel
P9	STAT1	2	614162	c.1030A>G	p.Lys344Glu	Missense	Heterozygous	AD			Deleterious (0)	probably_damaging (0.977)	Novel
P10	STAT1	2	614162	c.397A>G	p.Thr133Ala	Missense	Heterozygous	AD	rs1397948697		Tolerated (0.8)	benign (0.002)	Novel
P11	PLCG2	16	614878	c.2534_2545del	p.Leu845_Leu848del	In frame deletion	Heterozygous	AD					Novel
P12	ADA	20	102700	c.320T>C	p.Leu107Pro	Missense	Compound Het	AR	rs121908739	0.000074	Deleterious (0)	probably_damaging (0.999)	(33)
P12	ADA	20	102700	c.1A>G	p.Met1Val	Start loss	Compound Het	AR	rs1363043396				Novel
**MUTATIONS IN GENES NOT INCLUDED IN THE PID CLASSIFICATION (UNEXPECTED FINDINGS)**													
P13	SKIV2L	6	614602	c.2203-1G>A		Splicing defect	Compound Het	AR	rs144714933	0.000008			(34)
P13	SKIV2L	6	614602	c.3187C>T	p.Arg1063X	Stop gained	Compound Het	AR	rs138923214	0.000129			(35, 36)
P14	MMACHC	1	277400	c.271dupA	p.Arg91LysfsX14	Frameshift	Homozygous	AR					(37)
P15	SLC27A4	9	608649	c.1320_1321insC	p.Gly442ArgfsX2	Frameshift	Homozygous	AR					Novel
P16	DSG1	18	615508	c.1A>G	p.Met1Val	Start loss	Compound Het	AR	rs1388337318				Novel
P16	DSG1	18	615508	c.2569C>T	p.Arg857X	Non-sense	Compound Het	AR	rs756245217				Novel
P17	DNAI2	17	612444	c.546C>A	p.Tyr182X	Non-sense	Homozygous	AR	rs770946088				Novel
P18	SIX6	14	212550	c.86G>C	p.Arg29Pro	Missense	Homozygous	AR			Deleterious (0.02)	Benign (0.387)	Novel
P19	RECQL4	8	268400	c.2789_2812del	p.His930_Leu937del	In frame deletion	Homozygous	AR					Novel
**INCOMPLETE GENOTYPES**/**VARIANTS OF UNCERTAIN SIGNIFICANCE (VUS)**													
P20	UNC13D	17	608898	c.3224G>A	p.Arg1075Gln	Missense	Heterozygous	AR	rs377594755	0.000197	Deleterious (0.01)	Possibly_damaging (0.892)	Novel
P21	RAG2	11	603554	c.1324G>A	p.Ala442Thr	Missense	Heterozygous	AR	rs763513886	0.000008	Deleterious (0)	Probably_damaging (0.998)	Novel
P22	PLCG2	16	614878	c.2393A>G	p.Asn798Ser	Missense	Heterozygous	AD	rs117077093	0.000676	Deleterious (0.05)	Probably_damaging (0.998)	Novel
P23	TRAF3	14	614849	c.718G>A	p.Val240Ile	Missense	Heterozygous	AD	rs200118821	0.000082	Tolerated (0.15)	Benign (0.01)	Novel
P24	NOD2	16	266600	c.2753C>A	p.Ala918Asp	Missense	Heterozygous	AR, AD	rs104895452	0.000395	Deleterious (0)	Probably_damaging (0.997)	(38)

*AD, autosomal dominant; AR, autosomal recessive; Chr, chromosome; IUIS, international union of immunological societies; MAF, minor allele frequency; SIFT, sorting intolerant from tolerant*.

Three patients (P1, P2, and P3) had mutations in genes predominantly causing antibody deficiencies.

P1 is a 15-year-old male referred to our center at the age of 4 due to recurrent viral infections (rubella, EBV, cytomegalovirus), growth delay, lymphadenopathies, and chronic ear infections. The patient further developed a large bowel lymphoma, and presented an ALPS-like phenotype. Previous Sanger sequencing studies of the *FAS* and *XIAP* genes were negative. CES revealed a heterozygous mutation in the *PIK3R1* gene, responsible for activated PI3K delta syndrome type 2 (APDS2). The mutation, c.1425+1G>A, is the one most often described in this syndrome (22, 23). Identification of this mutation allowed the patient to enter a clinical trial for a selective PI3K delta inhibitor, but he had to discontinue after several weeks due to severe adverse effects. Unfortunately, he ultimately died at the age of 18 years.

Patients P2 and P3 had common variable immune deficiency (CVID)-like antibody deficiencies. P2 is a 57-year-old woman who reported recurrent upper respiratory tract infections since the age of 14 years, which continued despite intravenous immunoglobulin (IVIG) treatment. She also experienced acute diarrhea due to *Aeromonas Hydrophila*, granulomatous lymphadenopathies, hepatosplenomegaly, long periods of fever with elevated acute phase reactants, hypogammaglobulinemia, central pancytopenia, and granulomatous-lymphocytic interstitial lung disease (GLILD). P3 showed the first symptoms at the age of 3. She had episodes of EBV infection, autoimmune pancytopenia, hypogammaglobulinemia, pathological axillary lymphadenopathies, and hepatosplenomegaly. Previous genetic studies ruled out *FAS* and *PIK3R1*mutations. In both patients, heterozygous mutations were found in *TNFRSF13B* (the gene that codes for TACI). P2 had one of the most common missense mutations associated with CVID, c.310T>C/p.Cys104Arg (24, 25), and P3 had the c.260T>A/p.Ile87Asn mutation (26, 27). It has been demonstrated that both these mutations impair the capacity of TACI ligation to activate the NFκB pathway (27). TACI mutations are not considered to be fully-penetrant causal mutations, as most carriers show no disease, but up to 10% of CVID patients bear a mutation in TACI (compared to approximately 1% of the healthy population), which supports a role of TACI mutations in CVID or CVID-like disorders. Therefore, in P2 and P3 heterozygous TACI mutations cannot be considered causal by itself, but a predisposing factor in combination with other genetic and/or environmental factors.

P4 is an 18-year-old male who, since the age of 14, had experienced severe Crohn's-like gut inflammation and arthritis. He had a maternal family history of autoimmunity, and Crohn's disease on the paternal side. Mutations in *XIAP* and *FOXP3* had been ruled out prior to this study. CES revealed a hemizygous missense mutation in *IKBKG* (the gene that codes for NEMO), c.169G>A/p.Glu57Lys. *IKBKG* hypomorphic mutations, which reduce but do not abolish NF-κB activation, have been identified in male patients with a spectrum of X-linked clinical phenotypes, ranging from anhidrotic ectodermal dysplasia (EDA) with immunodeficiency (EDA-ID) to immunodeficiency without EDA. *IKBKG* loss-of-function mutations are lethal in males and cause incontinentia pigmenti (IP) in females (39). Our patient did not have EDA and the main clinical manifestation was gastrointestinal inflammation. He did not show hypogammaglobulinemia or relevant infections, 2 clinical features usually seen in typical EDA-ID patients (39). The *IKBKG* p.Glu57Lys mutation has been previously described in 2 unrelated patients, 1 with EDA-ID (40), and 1 with immunodeficiency without EDA (28). The latter study demonstrated that *IKBKG* p.Glu57Lys leads to specific immunological defects *in vitro* (28). Interestingly, this mutation has also been associated with a mild form of IP (29, 41). Given the relatively high frequency of p.Glu57Lys in the general population (MAF = 0.001), this mutation if pathogenic should be considered to have low penetrance.

P5 is 10-year-old girl who experienced recurrent atypical mycobacterial infections mainly in skin and gut since the age of 4. She also had hypertelorism, craniosynostosis, and scoliosis. Low Th17 cell counts and increased IgE levels pointed to mutations in genes responsible for hyper IgE syndrome, and indeed, CES revealed a heterozygous missense mutation in *STAT3* (c.1144C>T/p.Arg382Trp). This mutation, located in the DNA-binding domain of the protein, is one of the most frequent *STAT3-*dominant negative mutations described (30, 31).

P6, a 2-year-old boy born of consanguineous parents, presented with enteropathy, bacterial infections, and growth delay at the age of 10 months. Based on a strong suspicion of PID, the attending pediatricians sent us a sample for inclusion in our genetic study, with no previous laboratory determinations. We found a novel hemizygous truncating mutation in *XIAP* (c.888_892del/p.Lys299LeufsX9), causing type 2 X-linked proliferation syndrome (XLP-2) (42), which perfectly fit the patient's clinical phenotype.

P7 is a boy referred to our hospital at the age of 4 months due to severe osteomyelitis caused by *Salmonella spp*. Functional tests revealed a normal respiratory burst test, but *in vitro* IFNγ and IL-12 production was impaired. Surprisingly, CES revealed a hemizygous mutation in *G6PD*, c.934G>C/p.Asp312His, reported to be responsible for class III G6PD deficiency. G6PD deficiency is the most common genetic cause of chronic hemolytic anemia (43). To date, nearly 200 *G6PD* mutations have been identified (44, 45), all of them causing a more or less marked *G6PD* deficiency, but preserving some residual G6PD activity in red cells (complete absence of G6PD activity would be lethal) (43). In the original descriptions, each *G6PD* variant was assigned to a class defined on the basis of residual enzymatic activity and clinical manifestations. The p.Asp312His mutation, also known as the Seattle-like variant, is a class III variant in which enzymatic activity is about 15% of normal (activity associated with class III ranges from 10 to 60% of normal activity) (46). Our patient did not have anemia or other cytopenias, although he showed reduced G6PD activity in red cells. Therefore, he was currently asymptomatic in terms of hemolytic anemia, which is not surprising as class III variants are considered to have incomplete penetrance (47). Furthermore, although severe G6PD deficiency can be a phenocopy of chronic granulomatous disease (48), that would not be the case of the p.Asp312His mutation associated with a mild form of G6PD deficiency. As was mentioned, our patient had a normal respiratory burst test and G6PD activity in granulocytes was within the normal range. In summary, this patient has a *G6PD* mutation that does not fully explain the clinical phenotype, and he remains under study.

In patients P8, P9 and P10, all showing a predominant clinical phenotype of chronic fungal infections since early ages, we found heterozygous mutations in *STAT1*. Heterozygous gain-of-function (GOF) mutations in *STAT1* are the most common genetic cause of chronic mucocutaneous candidiasis (CMC) (49, 50). Almost all reported patients present with fungal infections (mainly candida), but also with a wide range of other clinical features, including bacterial infections primarily affecting the respiratory tract (51). The STAT1 mutations in P8 (c.1060C>G/p.Leu354Val) and P9 (c.1030A>G/p.Lys344Glu) were novel and had occurred *de novo* in the patients. Both mutations are located in the DNA-binding domain of the protein, which, together with the coiled-coil domain (CCD), are the 2 main STAT1 domains where GOF mutations are located (49, 51). A mutation affecting the same residue of STAT1 (p.Leu354Met) as in P8 has been described in a patient with CMC (51). Patient P10 also showed a novel STAT1 mutation (c.397A>G/p.Thr133Ala) inherited from his asymptomatic mother. The Thr133Ala variant is located outside of, but very close to the CCD, which spans residues 136 to 317. The patient has 2 siblings who are also carriers of the mutation and who have also had CMC episodes since an early age. The CMC episodes of all 3 affected children resolved with the oral antifungal fluconazole, and all of them remain symptom-free to date.

P11 is a 12-year old girl with very early-onset severe skin and eye inflammation and recurrent respiratory infections (due to profound hypogammaglobulinemia), leading to the development of multiple bronchiectasis. CES revealed a novel, *de novo* heterozygous mutation in *PLCG2*, consisting of a 12-bp in-frame deletion in exon 24, leading to the loss of 4 amino acids (c.2534_2545del/p.Leu845_Leu848del). The deletion was located in the protein region encoding autoinhibitory domains. Heterozygous GOF mutations affecting the autoinhibitory domains of PLCG2 have been associated with 2 different syndromes: PLAID (phospholipase Cγ2-associated antibody deficiency and immune dysregulation) and APLAID (autoinflammation and phospholipase Cγ2-associated antibody deficiency and immune dysregulation) (52, 53). Both syndromes are autoinflammatory-related disorders that typically show sinopulmonary infections caused by a humoral defect. The main difference between them is that APLAID patients do not have cold-induced urticaria, as occurs in patients with PLAID syndrome (54). The 2 phenotypes seem to be related to the type of mutation: PLAID is a consequence of large genomic *PLCG2* deletions (52), and the only 2 families reported with APLAID carried missense mutations (S707Y and L848P) (53, 55). However, since very few families with PLAID or APLAID have been described so far, this distinction must be considered with caution. In fact, P11 had a clinical phenotype compatible with APLAID syndrome, whereas the mutation was a small in-frame deletion, a type of mutation that has not yet been reported in APLAID. Immunological and functional studies are being conducted to investigate the pathogenicity of the *PLCG2* p.Leu845_Leu848del mutation and determine the functional abnormalities related with activation of the NLRP3 inflammasome.

P12 is a 15-year old boy who presented with Hodgkin lymphoma and hypogammaglobulinemia (with low B and NK cell counts) at the age of 8 years. He experienced recurrent bacterial respiratory tract infections, including pneumonia. At the time of the Hodgkin lymphoma diagnosis, concomitant EBV infection was detected. This led us to perform direct sequencing of the *SH2D1A* (SAP), *XIAP*, and *PIK3R1* genes, with negative results. CES revealed a compound heterozygous mutation in the *ADA* gene, consisting of a previously reported missense mutation (c.320T>C/p.Leu107Pro) and a novel mutation affecting the start codon (c.1A>G/p.Met1Val). It has been demonstrated that p.Leu107Pro is a loss-of-function mutation that retains <0.01% of wild-type activity (56). Complete ADA deficiency is a well-known cause of severe combined immunodeficiency (SCID) but, clearly, our patient did not have a SCID phenotype. Hypomorphic mutations in the *ADA* gene can lead to a different immunodeficiency with a variable phenotype including signs of immune dysregulation (56, 57). As our patient fit in the category of late-onset ADA deficiency, we hypothesize that the novel p.Met1Val is a hypomorphic mutation allowing some ADA activity and thereby, rescuing our patient from a more severe phenotype (the functional effect of the p.Met1Val mutation is under study).

### Patients With Mutations in Genes Not Included in the PID Classification: Unexpected Findings

After the first step in the analytical strategy, focused on PID genes included in the IUIS classification, all patients with negative findings underwent a second analysis including the remaining genes of the clinical exome. Seven additional patients were diagnosed in this second analysis. As all patients were included in this study based on a suspicion of PID, most of the following findings were unexpected and interesting to consider in the differential diagnosis of suspected PID. The clinical and laboratory data of all patients described are summarized in Tables 3, 4.

#### *SKIV2L*—Trichohepatoenteric Syndrome 2 (OMIM #614602)

P13 is a 5-year-old boy who presented with acute gastroenteritis, *Enterococcus faecalis* bacteremia, and dehydration at the age of 8 months. Thereafter, he had episodes of viral disease that severely affected his enteropathy. He also experienced catheter-associated bacteremia caused by *Staphylococcus epidermidis*. The patient had short stature and psychomotor delay. Immunological laboratory tests disclosed profound hypogammaglobulinemia, absence of a vaccine response, and low lymphocyte proliferation after PHA stimulation. Based on these findings, IVIG treatment was initiated. The immunodysregulation polyendocrinopathy enteropathy X-linked (IPEX) syndrome was initially suspected, but direct sequencing of *FOXP3* showed no mutations. A second CES analysis revealed a compound heterozygous mutation in *SKIV2L*, consisting of one mutation affecting the intron 18 consensus acceptor splice site (c.2203-1G>A) and another introducing a premature stop codon in exon 26 (c.3187C>T/p.Arg1063X). The parents were heterozygous carriers. Both these mutations have been recently reported in several patients (34–36). Homozygous or compound heterozygous mutations in *SKIV2L* lead to type-2 trichohepatoenteric syndrome (THES2), first described in 2012 as a rare congenital bowel disorder mainly characterized by intractable diarrhea (58). Fewer than 40 patients have been reported so far with this condition [excellently reviewed in Bourgeois et al. (36)], which is characterized by 9 main clinical signs: intractable diarrhea, hair abnormalities, facial dysmorphism, intrauterine growth restriction (IUGR), immunodeficiency, skin abnormalities, liver disease, congenital heart defects, and platelet anomalies. Accordingly, our patient presented a similar phenotype with low stature, neonatal enteropathy, absence of a vaccination response, intellectual disability, and extraordinarily brittle hair.

Generically, trichohepatoenteric syndrome can be caused by mutations in either *SKIV2L* (THES2) or *TTC37* (THES1), with very similar clinical manifestations (although patients with *SKIV2L* mutations seem more severely affected than those with *TTC37*). Whereas, *TTC37* deficiency is included in the IUIS classification of PIDs in the category of predominantly antibody deficiencies (59), *SKIV2L* deficiency is not present. We propose that *SKIVL2* deficiency be included in the next IUIS classification. The above-mentioned features and the defects in the B and T cell compartments seen in our patient and other reported THE patients (60), indicate that both *TTC37* and *SKIV2L* deficiency should be included in the category of combined immunodeficiencies with associated or syndromic features.

#### *MMACHC*—Methylmalonic Aciduria and Homocystinuria (OMIM #277400)

P14 had a family history of an older brother who died at the age of 4 months with a diagnosis of hemophagocytic lymphohistiocytosis (HLH) and pancytopenia. Hence, when he was referred to our center at the age of 1 month with a clinical phenotype of pancytopenia, bone marrow hemophagocytosis, and increased ferritin levels, familial HLH and X-linked lymphoproliferative syndrome (XLP-1, XLP-2) were both suspected. The patient showed no cytotoxicity or degranulation, and perforin expression was normal on flow cytometry. Following CES, we carefully analyzed genes related with HLH and XLP (*PRF1, UNC13D, STX11, STXBP2, SH2D1A, XIAP*), but no mutations were found. Other genes in the virtual PID panel were also normal. Finally, in the overall CES analysis, a homozygous insertion was detected in one nucleotide of the *MMACHC* gene (c.271dupA), leading to a truncated protein (p.Arg91LysfsX14). The non-consanguineous parents were heterozygous carriers. Biallelic mutations in *MMACHC* cause a disease referred to as methylmalonic aciduria and homocystinuria, which is a genetically heterogeneous disorder of cobalamin (vitamin B12) metabolism (61). Two distinct phenotypes of *MMACHC* (cobalamin C) deficiency have been defined in terms of age of onset. Early-onset patients present in the first year of life with non-specific systemic, neurological, and hematological abnormalities, and have a poor outcome. Late-onset patients usually show acute neurological deterioration after the age of 4 years, with a better outcome after treatment (62, 63). Our patient clearly fit in the early-onset group, which is concordant with his genotype, as c.271dupA is the most common related mutation in Europeans and is associated with severe early-onset disease (37, 64).

Although uncommon, HLH can be the initial presentation of *MMACHC* deficiency. Wu and collaborators reported on a 4-month-old patient initially diagnosed with HLH, who was later diagnosed with cobalamin C disease (cblC) (65). Therefore, early-onset cblC should be considered in the differential diagnosis of a patient with a clinical presentation of very early-onset infantile HLH. Our patient ultimately died despite the start of intravenous of vitamin B12 treatment. Tandem mass spectrometry-based newborn screening should include the detection of congenital cobalamin defects, including *MMACHC* deficiency, to allow the early diagnosis prior to the onset of symptoms.

#### *SLC27A4*—Ichthyosis Prematurity Syndrome (OMIM #608649)

P15 is a 44-year-old woman from a consanguineous family. She was referred to the Dermatology Department due to excessive skin inflammation since infancy. Dermatological findings lead to a suspicion of Netherton syndrome, an autosomal recessive disease caused by mutations in the *SPINK5* gene and characterized by congenital ichthyosis, bamboo hair, and atopic diathesis. Although mainly a skin disorder, Netherton syndrome was defined as a PID in 2009, when the group of Professor H. Ochs evaluated the immune system in a cohort of Netherton patients and found that almost all had reductions in memory B cell counts, a defective vaccine response, increased inflammatory cytokines, and low NK cytotoxicity (66). The initial CES analysis focused on the *SPINK5* gene, which was found to be completely normal. The differential diagnosis of Netherton syndrome includes genes causing inherited ichthyoses, PIDs with hyper IgE, atopic dermatitis, and seborrheic dermatitis, among other conditions (67, 68). The virtual PID panel analysis was negative, but analysis of the other related genes identified a homozygous truncating mutation in the *SLC27A4* gene (c.1320_1321insC/p.Gly442ArgfsX2). Biallelic mutations in *SLC27A4* (mainly known as FATP4) cause ichthyosis prematurity syndrome (IPS), an autosomal recessive disorder characterized by premature birth and neonatal respiratory complications, followed by lifelong ichthyosis with atopic manifestations (69). In patients with autosomal recessive congenital ichthyosis, IPS is a rare condition, occurring in <4% of cases (70). Although they are very similar conditions, there are some differences between Netherton syndrome and IPS, mainly related with the skin manifestations. Relevant to immunity, Netherton syndrome may involve recurrent infections whereas IPS does not. This and the immune defects initially reported by Professor H. Ochs indicate that immunological laboratory tests are important to confirm or rule out suspected Netherton syndrome.

#### *DSG1*—Erythroderma, Congenital, With Palmoplantar Keratoderma, Hypotrichosis, and Hyper IgE (OMIM #615508)

P16 is a female born of non-consanguineous Spanish parents who, since the first 48 h of life, presented with erythrodermic skin lesions co-occurring with bacterial infections, mainly skin infections due to *Staphylococcus aureus* and sepsis caused by *S. epidermidis*. The lesions increased in extension and predominantly affected the distal limbs and the perioral and periocular regions, causing uncontrollable itching. Laboratory findings showed marked eosinophilia, reaching a value of 18% and 3.3 × 10^9^ cells/L (normal range: 6–6.5%; 0–0.7 × 10^9^/L). Immunoglobulin levels were normal except for increased IgE (>2,000 KU/L). Immunophenotyping showed an abnormal T cell phenotype, with a predominance of memory/effector T cells. All these findings led to a differential diagnosis that initially included hyper IgE syndrome (*TYK2, STAT3, DOCK8*), Netherton syndrome (*SPINK5*), and Omenn syndrome (*RAG1, RAG2*). Previous Sanger sequencing of *RAG1* and *RAG2* showed no mutations, and the patient was included in the CES project. The first analysis of sequencing data (virtual PID panel) showed a heterozygous missense variant in *SPINK5* (c.2773G>C/p.Asp925His). This variant was not reported in the main databases (dnSNP, ExAC, gnomAD), and the computational predictions were discordant (Supplementary Table 6). The variant was classified as a VUS, and no other low-frequency variants were found in *SPINK5*. Netherton syndrome is caused by biallelic mutations in *SPINK5*, and all those reported are loss-of-function mutations (non-sense, indels, and splicing mutations) (71). Therefore, the diagnosis of Netherton syndrome was not supported by the genetic data. A subsequent analysis revealed 2 heterozygous mutations in *DSG1*: one was a start loss mutation (c.1A>G/p.Met1Val) and the other a non-sense mutation (c.2569C>T/p.Arg857X). *DSG1* encodes desmoglein 1, a major constituent of desmosomes, which have a crucial role in maintaining epidermal integrity and barrier function. Biallelic mutations in *DSG1* cause SAM (severe dermatitis, multiple allergies, and metabolic wasting) syndrome (OMIM#615508) (72). Both mutations found in our patient were private and her parents were heterozygous carriers. SAM syndrome closely resembles Netherton syndrome, including congenital ichthyosis, erythroderma, and severe atopic dermatitis (73). As occurred in our patient and is reported in Netherton syndrome, elevated serum IgE and absolute eosinophil counts are found in *DSG1-*deficient patients (74–76). Other laboratory immune defects have not been described to date. It was recently demonstrated that DSG1 inhibits skin inflammation by inhibiting the NF-kB signaling pathway (77). Consequently, *DSG1* deficiency is linked to inflammation and points to a crucial link between loss of epithelial barrier integrity and immunologic dysregulation (77).

#### *DNAI2*—Primary Ciliary Dyskinesia (OMIM #612444)

P17 is a 1-year old girl born of consanguineous parents. She had a sister who died in the first year of life due to an unspecified heart disease, and a 9-year-old brother with asthma, growth delay, and multiple respiratory infections. At the age of 1 month, the patient was referred to our hospital due to malignant pertussis. During the following 3 months she experienced 4 episodes of acute bronchitis, requiring 3 hospitalizations. At the age of 6 months she was admitted again to our hospital to study failure to thrive. At 18 months of age, she developed biphenotypic acute leukemia (i.e., acute leukemia with a single population of blasts co-expressing markers of 2 different lineages), which was treated with allogeneic cord blood hematopoietic stem cell transplantation. PID was suspected based on the family history, recurrent respiratory tract infections, and malignancy. No pathogenic variants were found in the virtual PID panel, but analysis of the other genes identified a homozygous non-sense mutation in the *DNAI2* gene (c.546C>A/p.Tyr182X). This gene codes for the dynein axonemal intermediate chain 2 (DNAI2), a protein belonging to the dynein intermediate chain family. *DNAI2* is highly expressed in trachea and testis and is involved in the dynein regulatory complex of respiratory cilia and sperm flagella motility (78). Biallelic mutations in DNAI2 have been described as causing primary ciliary dyskinesia (PCD) (79). PCD comprises a group of rare and genetically heterogeneous disorders characterized by defective ciliary motility. PCD is caused by biallelic mutations in more than 30 genes (the number is rapidly increasing) related with the structure and function of the cilia and flagella (80). Patients with these disorders have a history of neonatal respiratory distress and later, recurrent and chronic infections of the upper and lower respiratory tracts that can lead to bronchiectasis and a progressive decline in lung function. Other manifestations include organ laterality defects (*situs inversus*), congenital heart disease, and male infertility (81). As it can be seen, some clinical features of PCD can overlap with other conditions, such as cystic fibrosis and primary immunodeficiencies. Therefore, the differential diagnosis between PCD and PID may be difficult, especially in children. The non-sense homozygous mutation found in *DNAI2* perfectly explains the clinical phenotype of our patient. Family study showed that the parents were heterozygous carriers and the older brother was homozygous, in accordance with his clinical symptoms (asthma, growth delay, and multiple respiratory infections). It is likely that this mutation was the cause of their sister's early death due to a congenital heart disease.

#### *SIX6*—Optic Disc Anomalies With Retinal and/or Macular Dystrophy (OMIM #212550)

P18 is a 43-year old man born of consanguineous parents, who presented with a dual phenotype showing both ocular and immunological manifestations. The patient's brother had died at the age of 7 years due to complications related to an immunodeficiency phenotype (more specific information was not available). The pedigree revealed a high degree of consanguinity in the family, and 2 cousins (a girl and a boy) had the same ocular phenotype. The patient had congenital cataracts, strabismus, and color blindness, although the most severe defect was coloboma. He reported a history of multiple infections and had a low platelet count and IgA deficiency. Despite normal numbers of T, B and NK cells, the patient's B cell subpopulations were imbalanced, with increased numbers of naïve B cells and reduced class-switch memory B cells. Immunodeficiency with coloboma has been reported in CHARGE syndrome (82), but our patient did not show most of the typical manifestations of this syndrome (OMIM#214800). aCGH showed no abnormalities and the patient was included in the CES study. Results of the virtual PID panel analysis were negative, but in the subsequent analysis, a rare homozygous variant was identified in the *SIX6* gene (c.86G>C/p.Arg29Pro). This variant had not been previously described in general population. Biallelic mutations in the *SIX6* gene cause optic disc anomalies with retinal or macular dystrophy (OMIM #212550), an autosomal recessive condition characterized by iris coloboma, decreased or absent vision, retinal dystrophy, and a colobomatous optic disc, among other manifestations (83, 84). Segregation studies in the family showed that both parents were heterozygous carriers of the p.Arg29Pro mutation. However, functional studies should be performed to confirm the pathogenicity of this variant. Since the defects reported in patients with *SIX6* deficiency are restricted to ocular anomalies and 2 relatives have the ocular phenotype but not the immunodeficiency, we conclude that our patient's immunological changes were caused by other genetic factors, monogenic, oligogenic, or involving other more complex models (as has been proposed in CVID and in selective IgA deficiency).

#### *RECQL4*—Rothmund-Thomson Syndrome (OMIM #268400)

P19 is a 5-year old boy from a consanguineous Pakistani family. At the age of 6 months, he experienced severe diarrhea that lasted for 1 month. He had growth delay and a polymalformative syndrome with skeletal malformations and poikiloderma. He developed systemic cytomegalovirus infection affecting the lung, and invasive aspergillosis that was difficult to control. Laboratory data showed hypogammaglobulinemia, lymphopenia, and thrombocytopenia. The CD4/CD8 ratio was increased, with a predominance of memory cells within the CD8 compartment (59% of CD8^+^CD45RO^+^ T cells). The *in vitro* lymphoproliferation capacity was impaired after stimulation with pokeweed mitogen, anti-CD3 (also supplemented with IL-2), or PHA. However, lymphoproliferation was normal with PMA-ionomycin, thus indicating an upstream signaling defect. Based on the patient's syndromic features, the first genetic test was aCGH, which showed no significant abnormalities. The patient was then included in the CES study because of suspected combined immunodeficiency (CID). None of the genes responsible for CID included in the panel carried pathogenic mutations. At that time, a diagnosis of Rothmund-Thomson syndrome (RTS) was suggested by the clinical geneticists and, indeed, a homozygous mutation was found in the *RECQL4* gene. It consisted of an in-frame deletion of 24 bp (c.2789_2812del), leading to the loss of 8 amino acids of the protein (p.His930_Leu937del). This mutation, along with other in-frame *RECQL4* deletions/duplications, have been described as pathogenic and a cause of RTS (85). RTS is an autosomal recessive genodermatosis presenting in infancy with a characteristic facial rash (poikiloderma) and other manifestations, including short stature, skeletal abnormalities, ocular defects, premature aging, and a predisposition to cancer (86). Although immune defects are not among the classical features of this syndrome, a few RTS patients with immunological abnormalities have been reported. In the 1990s, 2 RTS patients with humoral immune deficiencies (involving hypogammaglobulinemia) were described (87, 88). In 2006, Broom et al. described a patient with CID and a classic RTS phenotype who experienced successful immune reconstitution following umbilical cord blood transplantation (89). Later, in 2010, De Somer at al presented a patient with RTS and immune deficiency who developed granulomatous skin lesions after primary varicella-zoster virus infection (90). In a more recent study, Smeets et al. shed light on the question of why mutations in *RECQL4* can lead to these immunological abnormalities. These authors found that Recql4 loss causes rapid bone marrow failure in mice, leading to profound disruption of immune development, including impaired B and T cell development (91).

Collectively, these data suggest a role of the DNA helicase RECQL4 in the development of the immune system, and indicate that screening for immune deficiency should be considered in patients with RTS.

### Incomplete Genotypes and Variants of Uncertain Significance

An inherent feature of NGS is generation of large amounts of data and identification of a myriad of genetic variants. The clinical significance of a variable fraction of these variants may be difficult to ascertain based on current knowledge; thus, they are referred to as variants of uncertain/unknown significance (VUS).

In all patients undergoing CES in this study and excluding the causal mutations described above (Table 4), we found a varying number of low-frequency (MAF>0.01) variants in the virtual PID panel, considered not to be a cause of the clinical phenotype (Supplementary Table 6, which does not include variants in Table 4). Most of these variants are rare polymorphisms with no clear functional impact, but others are monoallelic loss-of-function variants in recessive genes that are clearly unrelated to the patients' disease. Nonetheless, a potential role of some of these variants as modulators of the clinical phenotype cannot be excluded.

A few of these variants, located in genes related to the patients' phenotypes, drew our attention (Table 4).

A heterozygous missense variant in *UNC13D* (p.Arg1075Gln) was identified in P20, who presented with pancytopenia, hepatosplenomegaly, hemophagocytosis, and subcutaneous panniculitis-like T cell lymphoma at the age of 8 years. Elevated HLH biomarkers and absent cytotoxicity and degranulation supported the diagnosis of fHLH. However, the functional tests normalized after the episode. The role of monoallelic variants in HLH is currently a topic of great interest and debate (92). We also sequenced *UNC13D* intron 1 by Sanger because pathogenic mutations have been reported in several HLH patients with monoallelic mutations in the coding regions (93–95), but no relevant variants were found. The role of the *UNC13D* p.Arg1075Gln variant is under study.

P21 is a male with failure to thrive and a family history of a sister who died at the age of 6 months due to unknown reasons. The patient had a clinical phenotype of multiple lymphadenopathies, intermittent abdominal pain, persistent fever, and granulomatous hepatitis. Analyses showed lymphopenia and low platelet counts. We found a heterozygous missense variant at very low frequency in the *RAG2* gene (p.Ala442Thr), classified as likely pathogenic following the ACMG rules (96). The clinical phenotype of the patient fits well with those observed in some patients with biallelic *RAG1* or *RAG2* mutations (97), but in this case we found only 1 mutation.

P22 presented with recurrent fevers seriously affecting his quality of life, with no response to colchicine or IL-1 inhibitors. Since the age of 6 years, he has also experienced episodes of sudden loss of muscle tone and consciousness. We identified a heterozygous missense variant in the *PLCG2* gene (p.Asn798Ser), predicted to be pathogenic by SIFT and PolyPhen2. The prevalence of this variant population is low (but not very low), and it was inherited from his mother.

In P23, a girl with herpes simplex encephalitis, we found a low-frequency heterozygous missense variant in *TRAF3* (p.Val240Ile). TRAF3 deficiency (autosomal dominant) has been associated with a clinical phenotype limited to herpes simplex encephalitis resulting from impairment of the TLR3 response. Only 1 patient with this condition has been reported (98). Our variant shows a low frequency in the general population, SIFT and PolyPhen2 classified it as benign, and it was inherited from the patient's mother. DNA-sensing pathway responsiveness is under study to evaluate the functional impact of this *TRAF3* variant.

P24 presented with early-onset inflammatory bowel disease at the age of 7 months, together with hypogammaglobulinemia. We identified a low-frequency missense variant in the *NOD2* gene (p.Ala918Asp). *NOD2* variants have been repeatedly associated with Crohn's disease (99), and p.Ala918Asp is among the ones most strongly associated (38). It seems clear that this variant is not the cause of the full clinical phenotype of P24, but it may play a role in the patient's gastrointestinal manifestations.

### Genetic Findings Post-clinical Exome in Patients With Negative Results

Typically, patients with negative results on genetic testing remain as “genetically undiagnosed,” and if suspicion of a genetic defect persists, they are usually included in other genetic testing approaches over time. During the 3 years of this study, most patients with negative CES results have been analyzed by other methods, and some of them ultimately received a genetic diagnosis. In P25, P26, and P27, clinically diagnosed as having CVID, biallelic mutations were identified in the *LRBA* gene. In 2012, LRBA deficiency was described as a monogenic cause of CVID associated with autoimmunity (100). *LRBA* is not included in the TruSight One Sequencing Panel, likely because it was discovered as a cause of CVID after the panel was designed (Illumina announced launch of the TSO panel in October 2013). This is one of the main limitations of CES: it can lack relevant genes described after it was designed. Samples from P26 and P27 were included in a targeted panel of CVID genes and P25 underwent WES. Of note, in P25, we reported the first case of LRBA deficiency due to uniparental disomy (101). A similar situation occurred in P28, clinically diagnosed as having CVID, but later tested with a more specific PID panel, which detected a heterozygous loss-of-function mutation in *IKZF1*. Monoallelic mutations in *IKZF1* (encoding IKAROS) were described as a monogenic cause of CVID in 2016 (102), but earlier, in 2012, a single patient with *IKZF1* mutation was reported to have congenital pancytopenia (103). This gene is also absent from the TSO. P29 is a patient with autoinflammatory manifestations, and additionally, psychomotor and growth delay. Following CES, we performed aCGH, which revealed a deletion of 13Mb on chromosome 6 including the *TNFAIP3* gene. *TNFAIP3* haploinsufficiency causes an autoinflammatory syndrome (Behcet-like) (104), which in our patient occurred together with other developmental impairments due to this large deletion (105). CES does not perform well for detecting large genomic rearrangements and, indeed, this defect was not detected in the CES data analysis. P30, with a strong clinical suspicion of X-linked agammaglobulinemia (XLA), was included in CES because we found no mutations in *BTK* by direct sequencing. CES was also negative. Since suspicion of XLA persisted, we performed western blot analysis of the BTK protein, which revealed an absence of BTK. Finally, a large deletion affecting the *BTK* 5′UTR was identified. As CES is focused on the coding regions, it was unable to detect this deletion. This limitation is inherent to all the approaches based on sequencing only the coding regions (usually targeted gene panels and WES) and can only be overcome by using WGS (or other specific techniques to detect CNVs). Finally P31 presented with monostotic fibrous dysplasia of the lumbar spine and episodes of bacterial infections with a complicated course. After CES had yielded negative results, we performed aCGH, which provided no relevant findings. Ultimately, the patient was diagnosed with Gorham-Stout Disease (GSD), a rare disorder characterized by angiogenesis, lymphangiomatosis, and severe bone resorption (106). The exact cause of GSD is unknown and no environmental, immunological or genetic risk factors have been identified. Bone loss in GSD is accompanied by uncontrolled growth (proliferation) of lymphatic tissue. Lymphatic abnormalities could be related to the recurrent infections in P31.

## Concluding Remarks

In this study, CES was used to analyze samples from 61 patients with clinically suspected PID. Aside from detection of mutations in genes typically causing PID, CES enabled molecular identification of defects in genes that are not present in the IUIS classification or in specific PID panels, as well as identification of unsuspected molecular etiologies. These findings indicate that some cases of clinically suspected PID may ultimately not correspond to a “genuine” PID, as a number of rare diseases include immune-related symptoms in their typical or atypical presentations. Furthermore, they illustrate one of the advantages of CES over the use of more specific PID panels. In contrast, the main limitation of CES for genetic diagnosis of PIDs is the lack of a considerable number of PID-causing genes. There is a simple reason for this: most of the absent genes were discovered after the clinical exome panel was designed. Typically, clinical exome panels provided by companies are not easily customized. In contrast, specific tailor-made PID panels can be periodically updated and may be more comprehensive in terms of PID-causing genes. Nonetheless, WES is becoming the gold-standard technique for the diagnosis of genetic diseases, and it is replacing CES as an unbiased approach covering all coding regions of the genome. However, despite continuing decreases in the cost of this technique, it remains expensive and the data analysis is difficult to manage for some centers. Hence, CES may still be a good practical option for this purpose.

Overall, we obtained a positive genetic finding in 42% of patients including CES approach and the other diagnoses achieved by later use of other techniques. Several reasons may explain the lack of genetic diagnosis in 58% of patients; the most intuitive one is the limited number of genes included in CES. Probably the use of WES and WGS would increase diagnostic rate but still a percentage of patients would remain undiagnosed. Very recently, Yska and collaborators systematically reviewed the diagnostic yield of NGS in genetically undiagnosed patients with PIDs and they found a broad range among the different studies (15–79%) (107). Several factors may explain this high variability, including methodological differences and the selected study populations. Other biological reasons like the existence of complex models of inheritance (e.g., oligogenic, polygenic, epigenetic) may difficult to achieve a definitive genetic diagnosis.

The number of VUS obtained in CES, WES and especially in WGS is also a challenging aspect inherent of NGS approaches. The ACMG guidelines suggest the management of VUS as follows: “*A variant of uncertain significance should not be used in clinical decision making. Efforts to resolve the classification of the variant as pathogenic or benign should be undertaken. While this effort to reclassify the variant is underway, additional monitoring of the patient for the disorder in question may be prudent*” (96). However, how to deal with VUS in a clinical setting is a continuous matter of debate (108, 109).

In summary, this study describes our experience in using CES as a tool for the genetic diagnosis of PIDs and discusses the expected and unexpected findings obtained. We believe it is of clinical interest to provide descriptions of patients with mutations in genes that are not included in the PID classification to aid in the differential diagnosis of some suspected PIDs. The cases described illustrate the heterogeneity and complexity encountered by professionals involved in the clinical management and genetic diagnosis of these disorders.

## Data Availability Statement

All relevant data from datasets generated for this study are included in the manuscript/Supplementary Files.

## Ethics Statement

The studies involving human participants were reviewed and approved by Ethics Review Board of Hospital Universitari Vall d'Hebron. Written informed consent was obtained from the patients or their legal representatives, according to the procedures of the Ethics Review Board of Hospital Universitari Vall d'Hebron.

## Author Contributions

FR performed the clinical exome sequencing and bioinformatics data analysis, and wrote a part of the manuscript. CF-J performed the immunological analyses, collected data from all patients, and wrote a part of the manuscript. MM-G and MG-P performed immunological analyses and collected patient data. AA-C and LM provided technical support for sample collection and the sequencing process. FV made substantial contributions to the study design and provided technical support in the sequencing process. IC, CS, NB-R, and MF-C contributed to the data analysis and interpretation of variants. AM-N, JR, VG-P, XS, II, JS-P, JT, CC, JV, RP-B, and PS-P clinicians in charge of patient care, were involved in management of the patients and collecting clinical data. RC performed the genetic analysis and was responsible for designing the study, writing the manuscript, and approving the final draft. All authors reviewed the manuscript and contributed to the final draft.

### Conflict of Interest

The authors declare that the research was conducted in the absence of any commercial or financial relationships that could be construed as a potential conflict of interest.
