# Supplementary material for: Expanding the Clinical and Genetic Spectra of Primary Immunodeficiency-Related Disorders With Clinical Exome Sequencing: Expected and Unexpected Findings
Source: Front Immunol. 2019 Oct 1;10:2325. doi: 10.3389/fimmu.2019.02325 (PMC6797824; doi:10.3389/fimmu.2019.02325)
Supplement: Supplementary file 1 [file Table_1.DOCX]

**Supplementary Table 1**. Data represents the mean of the NGS Quality Control Metrics of all runs.

Miseq platform (3 samples per run)

| **Number of runs = 20** |  |  |  |
| --- | --- | --- | --- |
| **Cluster PF (%)** | **Reads PF** | **Density (K/mm^2^)** | **%≥Q30** |
| 88.58 | 23,675,292 | 1,183 | 89.68 |

Nextseq platform (36 samples per run)

| **Number of runs = 2** |  |  |  |
| --- | --- | --- | --- |
| **Cluster PF (%)** | **Reads PF** | **Density (K/mm^2^)** | **%≥Q30** |
| 86.53 | 96,727,198 | 151 | 80.55 |
