# Supplementary material for: Expanding the Clinical and Genetic Spectra of Primary Immunodeficiency-Related Disorders With Clinical Exome Sequencing: Expected and Unexpected Findings
Source: Front Immunol. 2019 Oct 1;10:2325. doi: 10.3389/fimmu.2019.02325 (PMC6797824; doi:10.3389/fimmu.2019.02325)
Supplement: Supplementary file 2 [file Table_2.DOCX]

**Supplementary Table 2**. Individual sample general sequencing data.

| **Patient** | **Aligned Reads (%)** | **Bases  Q-score>30 (%)** | **Target Coverage at 20X (%)** | **Uniformity of  Coverage (%)** | **Mean Region Coverage Depth (X)** |
| --- | --- | --- | --- | --- | --- |
| **P1** | 95.3 | 91.3 | 87.4 | 90.8 | 81.1 |
| **P2** | 99.8 | 94.9 | 91.7 | 95.5 | 64.1 |
| **P3** | 53 | 51.7 | 85.3 | 95.8 | 53.8 |
| **P4** | 98.4 | 92.8 | 89.7 | 93.2 | 78.6 |
| **P5** | 94.9 | 87.9 | 77.2 | 88.6 | 62.9 |
| **P6** | 95.8 | 94.7 | 88.9 | 91.9 | 81.9 |
| **P7** | 88.7 | 93.4 | 83.9 | 88.9 | 75.5 |
| **P8** | 83.8 | 94.7 | 92.1 | 96.3 | 67.2 |
| **P9** | 89.1 | 90.2 | 80.1 | 87 | 72.5 |
| **P10** | 95.6 | 94.8 | 85.6 | 91.7 | 71.1 |
| **P11** | 95.8 | 95.4 | 92 | 94.1 | 80.9 |
| **P12** | 99.1 | 86.4 | 97.4 | 96.7 | 111.8 |
| **P13** | 98.6 | 94 | 85 | 91 | 70 |
| **P14** | 90.1 | 93.2 | 79.8 | 94.4 | 48.4 |
| **P15** | 99.7 | 85 | 97.1 | 96.4 | 110.8 |
| **P16** | 98 | 94.5 | 78.1 | 91.1 | 55.2 |
| **P17** | 95.3 | 95.6 | 71.6 | 95.1 | 38.4 |
| **P18** | 86.6 | 89.1 | 93.3 | 95.8 | 78.7 |
| **P19** | 96.5 | 95 | 77.4 | 95.6 | 44.8 |
| **P20** | 98.9 | 96.8 | 84.9 | 95.4 | 51.9 |
| **P21** | 95.7 | 88.3 | 88.8 | 90.9 | 85.8 |
| **P22** | 87 | 95.2 | 93.9 | 96.1 | 77.5 |
| **P23** | 87.1 | 91 | 94.2 | 94.8 | 93.4 |
| **P24** | 99.7 | 83 | 97.2 | 96.6 | 105.5 |
| **P25** | 98.8 | 95.2 | 93 | 95.5 | 78.3 |
| **P26** | 98.4 | 95.5 | 93.6 | 95.9 | 79.5 |
| **P27** | 98 | 94.6 | 64.8 | 91.4 | 38.7 |
| **P28** | 86.7 | 93.8 | 86.9 | 94.3 | 62.2 |
| **P29** | 96.5 | 95 | 90 | 93.5 | 77.1 |
| **P30** | 86.4 | 92.4 | 94.8 | 95.4 | 92.2 |
| **P31** | 98.6 | 93.4 | 95.4 | 94.9 | 100.6 |
| **P32** | 99 | 96.7 | 92.4 | 95.1 | 78.1 |
| **P33** | 94.9 | 95.7 | 83.2 | 94.7 | 53 |
| **P34** | 95.5 | 94.5 | 54.9 | 88.2 | 32 |
| **P35** | 95.5 | 88.8 | 85 | 90.2 | 75.3 |
| **P36** | 52.7 | 51.9 | 90.1 | 94.7 | 72 |
| **P37** | 88.1 | 91.5 | 88.6 | 92.5 | 77.8 |
| **P38** | 86.5 | 88.7 | 97.1 | 96.5 | 107.5 |
| **P39** | 87 | 88.9 | 96.6 | 95.9 | 106.9 |
| **P40** | 86.4 | 90.8 | 69.9 | 82.3 | 61 |
| **P41** | 85.2 | 88.7 | 92.6 | 96 | 70.3 |
| **P42** | 86.2 | 88.9 | 90.2 | 95.5 | 69.7 |
| **P43** | 81.9 | 87.7 | 97.4 | 97.1 | 105.2 |
| **P44** | 85 | 89.2 | 95.4 | 97.4 | 75 |
| **P45** | 83.4 | 90.1 | 96.3 | 97 | 87.8 |
| **P46** | 99.6 | 77.1 | 96.8 | 97.1 | 90.2 |
| **P47** | 99.6 | 82.4 | 89.8 | 91.6 | 89.1 |
| **P48** | 99.7 | 82.6 | 95.3 | 96.1 | 86.1 |
| **P49** | 99.5 | 87.9 | 97.6 | 97.1 | 108.7 |
| **P50** | 99.7 | 84.8 | 94.2 | 96.2 | 77.6 |
| **P51** | 99.4 | 89.4 | 92 | 85.2 | 184.3 |
| **P52** | 99.6 | 77.7 | 96.4 | 96.4 | 97.4 |
| **P53** | 99.3 | 85.6 | 96.6 | 96.1 | 106.6 |
| **P54** | 99.7 | 84.2 | 97.3 | 96.4 | 119.4 |
| **P55** | 99.6 | 82.4 | 88.2 | 86.4 | 107.7 |
| **P56** | 99.7 | 83.9 | 93.9 | 95.6 | 83.1 |
| **P57** | 99.7 | 82.9 | 93.9 | 93.4 | 102.5 |
| **P58** | 99.9 | 93.6 | 84.8 | 90 | 62 |
| **P59** | 99.4 | 89.6 | 92 | 84.9 | 193.9 |
| **P60** | 99.9 | 94.2 | 89.4 | 95.7 | 52.1 |
| **P61** | 95.2 | 90.9 | 76.2 | 86.1 | 65.3 |
| **Mean** | **93.2** | **89.0** | **88.8** | **93.5** | **81.8** |
| **SD** | **9.4** | **8.4** | **8.6** | **3.6** | **28.4** |
| **Max** | **99.9** | **96.8** | **97.6** | **97.4** | **193.9** |
| **Min** | **52.7** | **51.7** | **54.9** | **82.3** | **32** |
