# Supplementary material for: Expanding the Clinical and Genetic Spectra of Primary Immunodeficiency-Related Disorders With Clinical Exome Sequencing: Expected and Unexpected Findings
Source: Front Immunol. 2019 Oct 1;10:2325. doi: 10.3389/fimmu.2019.02325 (PMC6797824; doi:10.3389/fimmu.2019.02325)
Supplement: Supplementary file 3 [file Table_3.DOCX]

**Supplementary Table 3**. Genes included in the virtual primary immunodeficiencies panel.

| ACP5 | CARD14 | CFP | FOXP3 | IL7R | NCF2 | RAB27A | STAT5B | TNFSF11 |
| --- | --- | --- | --- | --- | --- | --- | --- | --- |
| ACTB | CARD9 | CFTR | FPR1 | IRAK4 | NCF4 | RAC2 | STIM1 | TPP1 |
| ADA | CASP10 | CHD7 | G6PC3 | IRF7 | NCSTN | RAG1 | STK4 | TPP2 |
| ADAM17 | CASP8 | CIITA | G6PD | IRF8 | NFKB1 | RAG2 | STX11 | TRAC |
| AICDA | CCBE1 | CLCN7 | GATA2 | ITCH | NFKBIA | RANBP2 | STXBP2 | TRAF3 |
| AIRE | CD19 | CORO1A | GFI1 | ITGB2 | NHEJ1 | RFX5 | TAP1 | TRAF3IP2 |
| AK2 | CD247 | CR2 | HAX1 | ITK | NHP2 | RFXANK | TAP2 | TREX1 |
| AP3B1 | CD27 | CSF2RA | HMOX1 | JAK3 | NLRP12 | RFXAP | TAPBP | TTC37 |
| APOL1 | CD3D | CSF2RB | ICOS | KDM6A | NLRP3 | RMRP | TAZ | TYK2 |
| ATM | CD3E | CSF3R | IFIH1 | KMT2D | NOD2 | RNASEH2A | TBK1 | UNC13D |
| B2M | CD3G | CTC1 | IFNAR2 | KRAS | NOP10 | RNASEH2B | TBX1 | UNC93B1 |
| BCL10 | CD40 | CTLA4 | IFNGR1 | LAMTOR2 | NRAS | RNASEH2C | TCIRG1 | UNG |
| BLM | CD40LG | CTSC | IFNGR2 | LIG1 | ORAI1 | RNF168 | TCN2 | USB1 |
| BLNK | CD46 | CXCR4 | IGHM | LIG4 | OSTM1 | RNU4ATAC | TERC | VPS13B |
| BTK | CD55 | CYBA | IGLL1 | LPIN2 | PEPD | SAMD9 | TERT | WAS |
| C1QA | CD59 | CYBB | IKBKG | LYST | PIK3CD | SAMHD1 | TFRC | WIPF1 |
| C1QB | CD79A | DCLRE1C | IL10 | MAGT1 | PIK3R1 | SBDS | THBD | WRAP53 |
| C1QC | CD79B | DDX58 | IL10RA | MASP2 | PLCG2 | SEMA3E | TICAM1 | XIAP |
| C1R | CD81 | DKC1 | IL10RB | MCM4 | PLEKHM1 | SERPING1 | TINF2 | ZAP70 |
| C1S | CD8A | DNMT3B | IL12B | MEFV | PMS2 | SH2D1A | TIRAP | ZBTB24 |
| C2 | CEBPE | DOCK8 | IL12RB1 | MKL1 | PNP | SH3BP2 | TLR3 |  |
| C3 | CFB | ELANE | IL17F | NFKB1 | POLE2 | SLC29A3 | TMC6 |  |
| C4A | CFD | EXTL3 | IL17RA | MOGS | PRF1 | SLC35C1 | TMC8 |  |
| C4B | CFH | FADD | IL1RN | MS4A1 | PRKDC | SLC46A1 | TMEM173 |  |
| C5 | CFHR1 | FASLG | IL21 | MSH6 | PSEN1 | SMARCAL1 | TNFAIP3 |  |
| C6 | CFHR2 | FCGR3A | IL21R | MTHFD1 | PSENEN | SNX10 | TNFRSF11A |  |
| C7 | CFHR3 | FCN3 | IL2RA | MVK | PSMB8 | SP110 | TNFRSF13B |  |
| C8A | CFHR4 | FERMT3 | IL2RG | MYD88 | PSTPIP1 | SPINK5 | TNFRSF13C |  |
| C8B | CFHR5 | FAS | IRAK1 | NBAS | PTEN | STAT1 | TNFRSF1A |  |
| C9 | CFI | FOXN1 | IL36RN | NCF1 | PTPRC | STAT3 | TNFRSF4 |  |
