# Supplementary material for: Expanding the Clinical and Genetic Spectra of Primary Immunodeficiency-Related Disorders With Clinical Exome Sequencing: Expected and Unexpected Findings
Source: Front Immunol. 2019 Oct 1;10:2325. doi: 10.3389/fimmu.2019.02325 (PMC6797824; doi:10.3389/fimmu.2019.02325)
Supplement: Supplementary file 4 [file Table_4.DOCX]

**Supplementary Table 4**. Main clinical features of patients included in this study.

| **ID** | **Sex** | **Age at study (y)** | **Gene** | **Family**  **History** | **Consanguinity** | **Age of onset (y)** | **Syndromic features** | **Infections** | **Autoimmunity** | **Lymphoproliferation** | **Inflammation** | **Main clinical features, key words** |
| --- | --- | --- | --- | --- | --- | --- | --- | --- | --- | --- | --- | --- |
| P1 | M | 15 | PIK3R1 | - | - | 7 | - | V | - | Malign | Gut | IgA deficiency, immune dysregulation, growth delay, enteropathy, intestinal large bowel lymphoma |
| P2 | F | 57 | TNFRSF13B | - | - | 14 | - | B | - | Benign | Gut, lung | CVID, infections, GLILD, granulomas, adenopathies, pancytopenia |
| P3 | F | 8 | TNFRSF13B | √ | - | 3 | - | B, V | √ | Benign | - | EBV, pancytopenia, hepatosplenomegaly, hypogammaglobulinemia, ALPS-like, CVID-like |
| P4 | M | 18 | IKBKG | √ | - | 14 | - | - | - | - | Gut, joints | Crohn’s disease, joint affectation, CGD, NEMO deficiency |
| P5 | F | 10 | STAT3 | - | - | 4 | √ | B, F, Myc | - | - | - | Hyper IgE, atypical mycobacteria |
| P6 | M | 2 | XIAP | - | √ | 0,8 | √ | B | - | - | Gut | Enteropathy, bacterial infection, growth delay |
| P7 | M | 1 | G6PD | - | - | 0,3 | - | B | - | - | Bone | Osteomyelitis, *Salmonella* spp. |
| P8 | M | 30 | STAT1 | - | - | 0,0 | - | F | √ | - | Skin, mucosa | Dermatophytosis, oral candidiasis |
| P9 | F | 4 | STAT1 | - | - | 0,8 | - | F | - | - | Joints | CMC, polyarthritis, episcleritis, bronchiectasis |
| P10 | M | 6 | STAT1 | √ | √ | 4 | - | F | - | - |  | Familiar CMC |
| P11 | F | 12 | PLCG2 | - | - | 0,8 | √ | B | - | - | Skin, lungs | Agammaglobulinemia, severe cutaneous inflammation, bronchiectasis, B cell lymphopenia, growth delay |
| P12 | M | 15 | ADA | - | - | 8 | - | B | - | Malign |  | Hodgkin lymphoma, B cell deficiency |
| P13 | M | 5 | SKIV2L | - | - | 0,8 | √ | B | - | - | Gut | Inflammatory enteropathy, growth delay |
| P14 | M | 0,25 | MMACHC | √ | - | 0,1 | - | - | - | - | - | fHLH, XLP |
| P15 | F | 44 | SLC27A4 | - | √ | n.a. | - | - | - | - | Skin | Netherton syndrome |
| P16 | F | 0,3 | DSG1 | - | - | 0,3 | - | B | - | - | - | Erythroderma, Netherton syndrome, hyper IgE, eosinophilia |
| P17 | F | 1 | DNAI2 | √ | √ | 0,1 | - | V, F | √ | Malign | - | Recurrent bronchitis, biphenotypic leukemia, growth delay |
| P18 | M | 38 | SIX6 | √ | √ | n.a. | - | B | - | - | - | CID, Low IgA |
| P19 | M | 5 | RECQL4 | - | √ | 1 | √ | V, F | - | - | - | CVID, growth delay |
| P20 | M | 11 | UNC13D | √ | - | 8 | - | V | - | Malign | Gut | Pancytopenia, hepatosplenomegaly, hemophagocytosis, panniculitic T cell lymphoma |
| P21 | M | 13 | RAG2 | √ | - | 6 | √ | - | - | Benign | Systemic | Persistent fever, intermittent abdominal pain, granulomatous hepatitis |
| P22 | M | 4 | PLCG2 | √ | √ | 1 | - | V | - | - | Skin | Periodic fever, skin rash |
| P23 | F | 11 | TRAF3 | - | - | 11 | - | V | - | - | - | Herpes Zoster, VZV meningoencephalitis |
| P24 | M | 2 | NOD2 | - | - | 0,6 | - | - | - | - | Gut | Early onset colitis |
| P25 | F | 8 | LRBA | √ |  | 0,7 |  | B, V, F | √ | Malign | Gut | EBV, lymphoproliferation, autoimmunity, infections, dysregulation, enteropathy, autoimmune cytopenia |
| P26 | F | 11 | LRBA | - | - | 2 | - | V | √ | Benign | - | ALPS |
| P27 | F | 7 | LRBA | - | - | 7 | - | B | √ | Benign | - | Antibody deficiency, autoimmunity, lymphoproliferative syndrome |
| P28 | F | 14 | IKZF1 | √ | - | 5 | √ | B | - | - | - | Agammaglobulinemia, neurological delay |
| P29 | M | 9 | 13 Mb del cr.6 | √ | √ | 0,0 | √ | B, V | - | - | - | Neutrophilic dermatosis, oral and genital aphthae, growth delay |
| P30 | M | 6 | BTK | - | - | 4 | - | B | - | - | - | Pneumonia, hypogammaglobulinemia, absence of B cells |
| P31 | M | 25 | Gorham-Staut disease | - | - | 21 | √ | B | - | - | Joints | Osteopenia, chylothorax, lymphopenia, *S.Aureus* bacteremia, septic arthritis |
| P32 | M | 11 | inconclusive | √ | - | 14 | - | V | - | Malign | Skin | CAEBV, T-lymphoproliferative syndrome associated with EBV, hemophagocytic syndrome |
| P33 | M | 0.17 | inconclusive | n.a. | n.a. | 0.17 | n.a. | - | - | - | Gut | Syndromic enteropathy, chronic diarrhea |
| P34 | F | 1 | inconclusive | - | - | 0.17 | - | B | - | - | - | Bacteremia due to S.pneumoniae, low acute phase reactants, neutropenia |
| P35 | F | 2 | inconclusive | - | - | 2 | - | Myc | - | - | - | Previously healthy |
| P36 | F | 2 | inconclusive | √ | - | 0.58 |  | Myc | - | - |  | BCGitis |
| P37 | F | 3 | inconclusive | - | - | 0 | - | V | - | - | - | HSV2 meningoencephalitis |
| P38 | M | 4 | inconclusive | - | - | 0.04 | √ | V | √ | Benign | - | Eczema, thrombocytopenia |
| P39 | M | 2 | inconclusive | - | - |  | - | B,V | √ | - | - | Thrombocytopenia |
| P40 | F | 11 | inconclusive | - | - | 12 | - | B | - | - | - | Meningitis caused by Listeria *monocytogenes* |
| P41 | M | 0.7 | inconclusive | √ | √ | 0.02 | √ | B, V | - | - | Gut | Coloboma in both eyes, chronic diarrhea, febrile peaks with increased acute phase reactants without infection |
| P42 | F | 0 | inconclusive | - | - | 0.16 | - | B | - | - | - | Necrotizing pneumonia due to *S.aureus* |
| P43 | F | 1 | inconclusive | - | - | 0.08 | - | V | - | Benign | Systemic | Hemophagocytosis, enterovirus encephalitis |
| P44 | M | 15 | inconclusive | - | - | 5 | √ | B,V | - | - | CNS, Gut | Behavior alterations, self-injury |
| P45 | M | 18 | inconclusive | - | - | 5 | - | - | √ | Benign | - | Adenopathies, mild hemolytic anemia, neutropenia and trombocytopenia |
| P46 | M | 5 | inconclusive | - | √ | 0.16 | √ | - | - | Benign | Skin | Erythroderma, SCID suspicion, hepato-dermal GVHD confirmed with biopsy, pancytopenia |
| P47 | M | 9 | inconclusive | - | - | 7 | - | - | √ | Benign | Skin, Joint, Gut, Kidney | Leukocytoclastic vasculitis, colitis, glomerulonephritis |
| P48 | F | 0.1 | inconclusive | - | √ | 0 | √ | - | - | Benign | - | Polymalformative syndrome, PID secondary to thymic aplasia, cardiopathy |
| P49 | M | 22 | inconclusive | - | - | 15 | - | B | - | - | Gut | Diarrhea |
| P50 | M | 15 | inconclusive | - | - | 9 | - | B | √ | Benign | - | Kikuchi disease, nephrotic syndrome, pneumonia, osteomyelitis, chronic otitis media, adenopathies, hypogammaglobulinemia |
| P51 | M | 17 | inconclusive | - | - | 15 | - | B | - | Malign | - | Liver transplantation, hyper IgM, absent response to polysaccharide vaccines |
| P52 | M | 18 | inconclusive | √ | - | 6 | - | B | - | - | - | Bronchiectasis |
| P53 | F | 0 | inconclusive | - | √ | 0.33 | - | V | - | Benign | Systemic | SCID phenotype, CMV encephalitis |
| P54 | F | 9 | inconclusive | - | - | 7 | √ | B | √ | Benign | Skin | Atopic dermatitis, absent response to polysaccharide vaccines, bronchiectasis |
| P55 | M | 8 | inconclusive | √ | - | 4 | - | B | - | - | - | Thrombocytopenia |
| P56 | M | 13 | inconclusive | √ | - | 5 | - | B,V | - | Benign | - | Hypogammaglobulinemia, CVID like, splenomegaly |
| P57 | M | 23 | inconclusive | - | - | infancy | √ | - | √ | - | - | Alopecia universalis |
| P58 | F | 4 | inconclusive | √ | - | 2.4 | - | B | - | - | - | *Exitus* |
| P59 | F | 40 | inconclusive | - | - | 5 | - | B, F | - | Benign | Skin, joints | CID, T-lymphoproliferative syndrome associated with EBV in skin, ENT mucosa and bone. Infections, bronchiectasis |
| P60 | M | 11 | inconclusive | - | - | 4 | - | B | - | - | - | - |
| P61 | M | 0.17 | inconclusive | √ | √ | 0,08 | √ | V,F | - | - | - | Absence of thymic shadow. SCID phenotype, Pneumocystis pneumonia |

n.a.: not available.

V: Virus, B: Bacteria, F: Fungi, Myc: Mycobacteria

ALPS: Autoimmune Lymphoproliferative Syndrome, BCG: Bacille Calmette-Guérin, CAEBV: Chronic active Epstein-Barr virus, CID: Combined Immunodeficiency, CGD: Chronic Granulomatous Disease, CMC: Chronic Mucocutaneous Candidiasis, CMV: cytomegalovirus, CNS: Central Nervous System, CVID: Common Variable Immunodeficiency, ENT: ear-nose-throat, EBV: Epstein-Barr Virus, fHLH: familial Hemophagocytic Lymphohistiocytosis, GLILD: granulomatous and lymphocytic interstitial lung disease, GVHD: Graft Versus Host Disease, HSV2: Herpes Simplex Virus type 2, SCID: Severe Combined Immunodeficiency, VZV: Varizella Zoster Virus, XLP: X-linked Lymphoproliferation
