# Supplementary material for: Expanding the Clinical and Genetic Spectra of Primary Immunodeficiency-Related Disorders With Clinical Exome Sequencing: Expected and Unexpected Findings
Source: Front Immunol. 2019 Oct 1;10:2325. doi: 10.3389/fimmu.2019.02325 (PMC6797824; doi:10.3389/fimmu.2019.02325)
Supplement: Supplementary file 5 [file Table_5.DOCX]

**Supplementary Table 5**. Laboratory data of patients included in this study.

| **ID** | **Sex** | **Age at study (y)** | **Gene** | **Hypogammaglobulinemia** | **Neutropenia** | **Lymphopenia** | **Thrombocytopenia** | **Immunophenotype** | **Functional tests** |
| --- | --- | --- | --- | --- | --- | --- | --- | --- | --- |
| P1 | M | 15 | PIK3R1 | - | - | - | - | Inverse CD4/CD8. No switched memory B cells | Low proliferation with anti-CD3, altered *in vivo* response to *Haemophilus influenzae* |
| P2 | F | 57 | TNFRSF13B | √ | √ | √ | √ | Low CD4 naïve T cells, low Tregs, low pre-switched and switched B cells | n.a. |
| P3 | F | 8 | TNFRSF13B | √ | √ | √ | √ | Low CD4 naïve T cells, low pre-switched and switched B cells | Normal proliferation, cytotoxicity and degranulation. |
| P4 | M | 18 | IKBKG | - | - | - | - | Increased DN gamma delta T cells (22%) | Normal respiratory burst test and cytotoxicity/degranulation assays |
| P5 | F | 10 | STAT3 | - | - | - | - | Low Tregs, low Th17 | Very low proliferation with anti-CD3 |
| P6 | M | 2 | XIAP | - | - | - | - | n.a. | n.a. |
| P7 | M | 1 | G6PD | - | - | - | - | Inverse CD4/CD8 ratio. Increased effector HLA-DR+ CD8+ T cells, low switched B cells | Very low proliferation with anti-CD3 and ConA, very low IFNγ and IL12 production |
| P8 | M | 30 | STAT1 | - | - | - | - | Myeloid DC >>> plasmacytoid DC | Negative *in vivo* response to Candidin. |
| P9 | F | 4 | STAT1 | - | - | - | - | Low CD4 naïve T cells, low Th17, low pre-switched B cells | Low proliferation with PHA |
| P10 | M | 6 | STAT1 | - | - | - | - | 3,4% DN TCR αβ T cells, increased Th1, low Th17, low Tregs | Low IL12 and IFNγ production |
| P11 | F | 12 | PLCG2 | - | - | - | - | T+ B- NK+ | Normal proliferation, normal respiratory burst test |
| P12 | M | 15 | ADA | √ | - | - | - | T+ B- NK- | Normal respiratory burst test |
| P13 | M | 5 | SKIV2L | √ | - | - | - | Normal | Low proliferation with PHA (normal to anti-CD3 and ConA), normal respiratory burst test |
| P14 | M | 0,25 | MMACHC | - | √ | - | √ | n.a. | Absent degranulation and cytotoxicity |
| P15 | F | 44 | SLC27A4 | - | - | - | - | Normal | Normal |
| P16 | F | 0,3 | DSG1 | - | - | - | - | Very low effector and memory T cells, low Th2, low Th1/Th17 | n.a. |
| P17 | F | 1 | DNAI2 | - | - | - | - | Normal | Low proliferation with PWM and PHA |
| P18 | M | 38 | SIX6 | - | - | - | √ | Low switched memory B cells | Low proliferation with anti-CD3 |
| P19 | M | 5 | RECQL4 | √ | - | √ | √ | Increased CD4/CD8 ration | Low proliferation with PWM,antiI-CD3 and ConA. |
| P20 | M | 11 | UNC13D | - | √ | √ | - | Normal | Alternate low/normal degranulation and cytotoxicity |
| P21 | M | 13 | RAG2 | - | - | √ | √ | Low T cells, low NK cells. | Normal proliferation, normal respiratory burst test |
| P22 | M | 4 | PLCG2 | - | √ | - | - | Normal | n.a. |
| P23 | F | 11 | TRAF3 | - | - | - | - | Normal | Absent IL-12 production, normal IFNγ production |
| P24 | M | 2,0 | NOD2 | - | - | - | - | Normal | Normal proliferation, normal respiratory burst test |
| P25 | F | 8 | LRBA | √ | √ | √ | - | T+ B- NK- | Low/absent degranulation and cytotoxicity |
| P26 | F | 11 | LRBA | - | - | - | - | 4.2% DN TCR αβ T cells, low effector and memory T cells, low Tregs | Low CD69 and CD40L expression |
| P27 | F | 7 | LRBA | √ | - | - | - | Low pre-switched and switched B cells | Low pneumococcal response |
| P28 | F | 14 | IKZF1 | √ | - | - | - | T+ B- NK+, low pre-switched and switched B cells | Negative ASLO |
| P29 | M | 9 | 13 Mb del cr.6 | - | - | - | - | Normal | Low proliferation with anti-CD3, normal TNFα production in response to LPS, normal degranulation and cytotoxicity |
| P30 | M | 6 | BTK | √ | - | - | - | T+ B- NK+ | n.a. |
| P31 | M | 25 | Gorham-Staut disease | - | √ | - | - | Low CD4 T cells, increased NK cells | Severe defect in IL12 production, altered respiratory burst test |
| P32 | M | 11 | inconclusive | - | √ | - | √ | Low pre-switched and switched B cells | Absent degranulation and cytotoxicity |
| P33 | M | 0.17 | inconclusive | n.d. | - | - | √ | Low Th1/Th17. Low pre-switched and switched B cells | Low proliferation with anti-CD3. Normal respiratory burst test |
| P34 | F | 1 | inconclusive | - | √ | - | - | Normal | Normal respiratory burst test. Low IFNγ production in response to LPS |
| P35 | F | 2 | inconclusive | - | √ | - | - | Normal | Absent IFNγ and IL-12 production |
| P36 | F | 2 | inconclusive | n.d. | √ | - | - | Normal | Absent IFNγ and IL-12 production |
| P37 | F | 3 | inconclusive | - | n.a. | n.a. | n.a. | Normal | Low response to TLR3 |
| P38 | M | 4 | inconclusive | √ | - | - | √ | Increased DN gamma delta T cells (7.3%) | Low proliferation with anti-CD3+IL-2 and with ConA. |
| P39 | M | 2 | inconclusive | - | - | √ | √ | Low B cells, low NK cells. | Very low proliferation with anti-CD3 |
| P40 | F | 11 | inconclusive | √ | n.a. | n.a. | n.a. | Increased effector and memory CD4+ T cells. Increased pre-switch B cells | Absent IL-12 and low IFNγ production. Low CD40L up-regulation after PMA+ionomycin stimulation |
| P41 | M | 0.7 | inconclusive | - | n.a. | n.a. | n.a. | Normal | Absent proliferation with anti-CD3 and with anti-CD3+IL-2. Very low CD40L up-regulation after PMA+ionomycin stimulation |
| P42 | F | 0 | inconclusive | - | n.a. | n.a. | n.a. | Very low Th1. Th17 in lower limit | Normal proliferation, normal respiratory burst test. Absent IFNγ production. Low IL-17 production. |
| P43 | F | 1 | inconclusive | - | n.a. | n.a. | n.a. | Normal | Normal proliferation, cytotoxicity and degranulation. |
| P44 | M | 15 | inconclusive | - | - | √ | - | Low T cells, inverse CD4/CD8 ratio | n.a. |
| P45 | M | 18 | inconclusive | √ | √ | - | √ | Increased CD4+ effector memory T cells. Increased Th1 cells. Low Th2 and Th17. Low switched B cells | n.a. |
| P46 | M | 5 | inconclusive | n.d. | √ | - | √ | T-B-NK- | Absent proliferation with PMA+Ionomycin, anti-CD3 and anti-CD3+IL2 |
| P47 | M | 9 | inconclusive | - | n.a. | n.a. | n.a. | Increased CD4/CD8 ratio, increased HLA-DR expression | n.a. |
| P48 | F | 0.1 | inconclusive | - | - | √ | - | Low T and B cells. Increased CD4+ effector memory T cells | Absent proliferation, cytotoxicity and degranulation. |
| P49 | M | 22 | inconclusive | √ | - | √ | √ | Low T and B cells. Very low naïve T cells. Low switched B cells. Increased transitional B cells. | Very low proliferation |
| P50 | M | 15 | inconclusive | √ | - | √ | - | n.a. | n.a. |
| P51 | M | 17 | inconclusive | √ | - | - | - | Increased CD4+ and CD8+ effector memory T cells. Low pre-switched and switched B cells | Low cytotoxicity and degranulation. |
| P52 | M | 18 | inconclusive | √ | n.a. | n.a. | n.a. | Increased CD4+ effector memory T cells. Low pre-switched and switched B cells, increased Tregs | n.a. |
| P53 | F | 0 | inconclusive | √ | √ | √ | √ | T-B-NK- | Very low proliferation with anti-CD3. Absent cytoxicity and degranulation. |
| P54 | F | 9 | inconclusive | √ | - | - | - | T+B+NKlow | Low proliferation with anti-CD3 and anti-CD3+IL-2. Normal cytoxicity and degranulation. Normal IFNγ production. |
| P55 | M | 8 | inconclusive | √ | - | - | √ | Low CD8 and increased ratio CD4/CD8. | n.a. |
| P56 | M | 13 | inconclusive | √ | √ | √ | √ | Low B and NK cells | Low proliferation with anti-CD3. Slightly reduced with anti-CD3+IL2 |
| P57 | M | 23 | inconclusive | √ | n.a. | n.a. | n.a. | Normal. Increased DN T cells (21%). Increased plasmablasts. | n.a. |
| P58 | F | 4 | inconclusive | √ | - | - | - | Normal | n.a. |
| P59 | F | 40 | inconclusive | √ | n.a. | n.a. | n.a. | Absent pre-switched and switched B cells. Low CD4 naive T cells, increased Th1 T cells. | Very low proliferation with PMA+ionomycin. Normal cytotoxicity |
| P60 | M | 11 | inconclusive | √ | - | - | - | Absent pre-switched and switched B cells | n.a. |
| P61 | M | 0.17 | inconclusive | √ | - | √ | - | Normal | Very low proliferation with PMA+ionomycin and with ConA. Low proliferation with PHA. Absent proliferation with anti-CD3. Normal cytotoxicity |

n.a.: not available. n.d.: not determined

ASLO: Anti-streptolysin O, ConA: Concanavalin A, DC: Dendritic cell, DN: Double negative, PHA: Phytohemagglutinin, PMA: Phorbol 12-myristate 13-acetate, PWM: Pokeweed, TLR3: Toll-like receptor type 3
