# Supplementary material for: Expanding the Clinical and Genetic Spectra of Primary Immunodeficiency-Related Disorders With Clinical Exome Sequencing: Expected and Unexpected Findings
Source: Front Immunol. 2019 Oct 1;10:2325. doi: 10.3389/fimmu.2019.02325 (PMC6797824; doi:10.3389/fimmu.2019.02325)
Supplement: Supplementary file 6 [file Table_6.DOCX]

**Supplementary Table 6**. Low-frequency variants of genes included in the virtual PID panel.

| **Patient** | **Gene** | **Chr** | **Genotype** | **Consequence** | **cDNA** | **Protein** | **dbSNP ID** | | **ExAC freq** | **Sift** | **PolyPhen** |
| --- | --- | --- | --- | --- | --- | --- | --- | --- | --- | --- | --- |
| P1 | MAN2B1 | 19 | het | missense | c.1444C>T (NM_000528.3) | p.Arg482Trp (NP_000519.2) | rs533428309 | | 0.000029 | deleterious (0.01) | benign (0.254) |
| P1 | JAK3 | 19 | het | missense | c.997C>T (NM_000215.3) | p.Pro333Ser (NP_000206.2) | rs199908476 | | . | tolerated (0.47) | benign (0.062) |
| P1 | IL7R | 5 | het | missense | c.214G>C (NM_002185.3) | p.Glu72Gln (NP_002176.2) | rs148001159 | | 0.000264 | tolerated (0.35) | benign (0.02) |
| P1 | CR2 | 1 | het | missense | c.1676G>A (NM_001006658.2) | p.Gly559Glu (NP_001006659.1) | rs143614333 | | 0.000775 | deleterious (0.01) | probably_damaging (0.954) |
| **Patient** | **Gene** | **Chr** | **Genotype** | **Consequence** | **cDNA** | **Protein** | **dbSNP ID** | | **ExAC freq** | **Sift** | **PolyPhen** |
| P2 | LRRC8A | 9 | het | missense | c.1111G>A (NM_019594.3) | p.Asp371Asn (NP_062540.2) | . | | . | deleterious (0) | probably damaging (0.985) |
| P2 | DOCK8 | 9 | het | missense | c.950G>A (NM_203447.3) | p.Arg317Gln (NP_982272.2) | rs191413750 | | 0.000074 | tolerated (0.18) | possibly damaging (0.781) |
| P2 | CD3G | 11 | het | missense | c.56G>A (NM_000073.2) | p.Gly19Asp (NP_000064.1) | rs146393315 | | 0.000634 | tolerated (0.11) | possibly damaging (0.462) |
| **Patient** | **Gene** | **Chr** | **Genotype** | **Consequence** | **cDNA** | **Protein** | **dbSNP ID** | | **ExAC freq** | **Sift** | **PolyPhen** |
| P3 | PLCG2 | 16 | het | missense | c.3493G>A (NM_002661.3) | p.Val1165Ile (NP_002652.2) | rs372557475 | | 0.000025 | tolerated (0.05) | probably_damaging (0.988) |
| P3 | SKIV2L | 6 | het | missense | c.3334C>T (NM_006929.4) | p.Leu1112Phe (NP_008860.4) | . | | . | deleterious (0) | probably_damaging (0.942) |
| P3 | DOCK8 | 9 | het | missense | c.950G>A (NM_203447.3) | p.Arg317Gln (NP_982272.2) | rs191413750 | | 0.000074 | tolerated (0.11) | benign (0.347) |
| P3 | PEPD | 19 | het | missense | c.751T>A (NM_000285.3) | p.Ser251Thr (NP_000276.2) | rs201572375 | | 0.000085 | tolerated (0.08) | benign (0.184) |
| P3 | VPS13B | 8 | het | missense | c.8645C>T (NM_017890.4) | p.Pro2882Leu (NP_060360.3) | rs145890213 | | 0.000955 | tolerated (0.11) | benign (0.043) |
| P3 | IL12B | 5 | het | missense | c.823G>A (NM_002187.2) | p.Val275Ile (NP_002178.2) | rs189324104 | | 0.000173 | tolerated (0.4) | benign (0.014) |
| **Patient** | **Gene** | **Chr** | **Genotype** | **Consequence** | **cDNA** | **Protein** | **dbSNP ID** | | **ExAC freq** | **Sift** | **PolyPhen** |
| P4 | GFI1 | 1 | het | missense | c.319C>G (NM_001127216.1) | p.Pro107Ala (NP_001120688.1) | rs149914857 | | 0.002583 | deleterious (0.01) | benign (0.239) |
| P4 | NLRP3 | 1 | het | missense | c.1312A>T (NM_001079821.2) | p.Thr438Ser (NP_001073289.1) | rs180177465 | | . | tolerated (0.05) | possibly_damaging (0.864) |
| P4 | IFNGR2 | 21 | het | missense | c.940G>A (NM_005534.3) | p.Asp314Asn (NP_005525.2) | rs369529404 | | 0.000041 | deleterious (0.01) | possibly_damaging (0.683) |
| P4 | NHP2 | 5 | het | missense | c.190G>A (NM_017838.3) | p.Val64Met (NP_060308.1) | rs79031130 | | 0.000428 | tolerated (0.05) | probably_damaging (0.973) |
| P4 | TAP2 | 6 | het | missense | c.1655A>G (NM_018833.2) | p.Glu552Gly (NP_061313.2) | rs151064661 | | 0.000206 | deleterious (0.01) | probably_damaging (0.988) |
| P4 | TLR3 | 4 | het | missense | c.2486G>A (NM_003265.2) | p.Arg829Lys (NP_003256.1) | . | | . | tolerated (0.08) | benign (0.356) |
| P4 | UNC13D | 17 | het | missense | c.2180G>A (NM_199242.2) | p.Arg727Gln (NP_954712.1) | rs747390615 | | 0.000635 | tolerated (0.47) | benign (0.064) |
| **Patient** | **Gene** | **Chr** | **Genotype** | **Consequence** | **cDNA** | **Protein** | **dbSNP ID** | | **ExAC freq** | **Sift** | **PolyPhen** |
| P5 | C3 | 19 | het | missense | c.2027C>T (NM_000064.2) | p.Thr676Met (NP_000055.2) | rs139945572 | | 0.000016 | tolerated (1) | benign (0.007) |
| P5 | KMT2D | 12 | het | missense | c.11006G>A (NM_003482.3) | p.Gly3669Asp (NP_003473.3) | rs760279764 | | 0.000017 | . | possibly_damaging (0.949) |
| P5 | KMT2D | 12 | het | missense | c.1967T>C (NM_003482.3) | p.Leu656Pro (NP_003473.3) | rs1169567206 | | . | . | benign (0) |
| **Patient** | **Gene** | **Chr** | **Genotype** | **Consequence** | **cDNA** | **Protein** | **dbSNP ID** | | **ExAC freq** | **Sift** | **PolyPhen** |
| P6 | ADA | 20 | het | missense | c.454C>A (NM_000022.2) | p.Leu152Met (NP_000013.2) | rs121908728 | | 0.000398 | deleterious (0.01) | probably_damaging (0.999) |
| P6 | DCLRE1C | 10 | het | missense | c.1560G>C (NM_001033855.1) | p.Lys520Asn (NP_001029027.1) | rs753955796 | | 0.000016 | tolerated (0.19) | benign (0.067) |
| P6 | PIK3CD | 1 | het | missense | c.1394C>T (NM_005026.3) | p.Thr465Met (NP_005017.3) | rs368722127 | | 0.001058 | tolerated (0.23) | possibly_damaging (0.741) |
| P6 | TYK2 | 19 | het | missense | c.1807G>A (NM_003331.4) | p.Val603Met (NP_003322.3) | rs140594440 | | 0.000281 | deleterious (0) | possibly_damaging (0.885) |
| P6 | UNC13D | 17 | het | missense | c.1616A>G (NM_199242.2) | p.Asp539Gly (NP_954712.1) | rs780794721 | | 0.000008 | tolerated (0.37) | benign (0.06) |
| **Patient** | **Gene** | **Chr** | **Genotype** | **Consequence** | **cDNA** | **Protein** | **dbSNP ID** | | **ExAC freq** | **Sift** | **PolyPhen** |
| P7 | CD247 | 1 | het | stop gained | c.301C>T (NM_198053.2) | p.Gln101Ter (NP_932170.1) | rs55729925 | | 0.000224 | . | . |
| **Patient** | **Gene** | **Chr** | **Genotype** | **Consequence** | **cDNA** | **Protein** | **dbSNP ID** | | **ExAC freq** | **Sift** | **PolyPhen** |
| P08 | BLM | 15 | het | missense | c.43C>T (NM_000057.2) | p.Arg15Cys (NP_000048.1) | rs148545569 | | 0.000229 | deleterious (0.02) | possibly_damaging (0.853) |
| P8 | C7 | 5 | het | stop gained | c.2188C>T (NM_000587.2) | p.Gln730Ter (NP_000578.2) | rs768866511 | | 0.000008 | . | . |
| P08 | IL12RB2 | 1 | het | missense | c.742G>C (NM_001559.2) | p.Asp248His (NP_001550.1) | rs545150086 | | 0.000016 | tolerated (0.14) | probably_damaging (0.991) |
| P8 | LIG4 | 13 | het | inframe deletion | c.1172_1174del (NM_206937.1) | p.Ser391del (NP_996820.1) | rs775835184 | | 0.000025 | . | . |
| P8 | PSTPIP1 | 15 | het | missense | c.1213C>T (NM_003978.3) | p.Arg405Cys (NP_003969.2) | rs201253322 | | 0.000469 | tolerated (0.11) | benign (0.105) |
| P08 | WIPF1 | 2 | het | frameshift | c.768dupC (NM_003387.4) | p.Ser257GlnfsTer6 (NP_003378.3) | . | | . | . | . |
| **Patient** | **Gene** | **Chr** | **Genotype** | **Consequence** | **cDNA** | **Protein** | **dbSNP ID** | | **ExAC freq** | **Sift** | **PolyPhen** |
| P9 | C1R | 12 | het | missense | c.647C>G (NM_001733.4) | p.Pro216Arg (NP_001724.3) | rs189155429 | | 0.000627 | tolerated (0.3) | possibly_damaging (0.58) |
| P9 | CD79A | 19 | het | missense | c.655G>C (NM_001783.3) | p.Gly219Arg (NP_001774.1) | rs1478564368 | | . | tolerated (0.29) | probably_damaging (0.991) |
| P9 | MEFV | 16 | het | missense | c.74A>G (NM_000243.2) | p.Lys25Arg (NP_000234.1) | rs924530771 | | . | deleterious (0) | probably_damaging (0.999) |
| **Patient** | **Gene** | **Chr** | **Genotype** | **Consequence** | **cDNA** | **Protein** | **dbSNP ID** | | **ExAC freq** | **Sift** | **PolyPhen** |
| P10 | UNC13D | 17 | het | missense | c.2341G>A (NM_199242.2) | p.Val781Ile (NP_954712.1) | rs149871493 | | 0.001269 | tolerated(0.47) | benign(0.001) |
| P10 | LPIN2 | 18 | het | missense | c.1159A>G (NM_014646.2) | p.Lys387Glu (NP_055461.1) | rs104895501 | | 0.00433 | tolerated(0.07) | benign(0.414) |
| P10 | KMT2D | 12 | het | missense | c.8774C>T (NM_003482.3) | p.Ala2925Val (NP_003473.3) | rs199547661 | | 0.001731 | . | benign(0.004) |
| P10 | KMT2D | 12 | het | missense | c.13274C>T (NM_003482.3) | p.Ala4425Val (NP_003473.3) | rs752531267 | | . | . | benign(0.067) |
| P10 | DKC1 | X | hom | missense | c.838A>C (NM_001363.3) | p.Ser280Arg (NP_001354.1) | rs146700772 | | 0.000319 | deleterious(0.03) | benign(0.008) |
| P10 | CSF3R | 1 | het | missense | c.1748G>A (NM_156039.3) | p.Arg583His (NP_724781.1) | rs148104401 | | 0.000594 | tolerated(0.88) | benign(0.003) |
| P10 | CSF3R | 1 | het | missense | c.1919C>T (NM_156039.3) | p.Thr640Ile (NP_724781.1) | rs121918426 | | 0.000541 | tolerated(0.99) | benign(0) |
| P10 | ATM | 11 | het | missense | c.3175G>A (NM_000051.3) | p.Ala1059Thr (NP_000042.3) | rs370282831 | | 0.000008 | tolerated(0.28) | benign(0.024) |
| **Patient** | **Gene** | **Chr** | **Genotype** | **Consequence** | **cDNA** | **Protein** | **dbSNP ID** | | **ExAC freq** | **Sift** | **PolyPhen** |
| P11 | LYST | 1 | het | missense | c.1727G>A (NM_000081.2) | p.Gly576Asp (NP_000072.2) | rs1240621396 | | . | deleterious (0.04) | probably_damaging (0.999) |
| P11 | RAG1 | 11 | het | missense | c.1864G>A (NM_000448.2) | p.Ala622Thr (NP_000439.1) | rs148380512 | | 0.000141 | deleterious (0) | probably_damaging (0.956) |
| P11 | TAP1 | 6 | het | missense | c.572T>C (NM_000593.5) | p.Leu191Pro (NP_000584.2) | rs142907576 | | 0.001506 | deleterious (0) | probably_damaging (0.999) |
| P11 | VPS13B | 8 | het | splice (donor) | c.580+1G>A (NM_017890.4) | . | . | | . | . | . |
| **Patient** | **Gene** | **Chr** | **Genotype** | **Consequence** | **cDNA** | **Protein** | **dbSNP ID** | | **ExAC freq** | **Sift** | **PolyPhen** |
| P12 | CHD7 | 8 | het | inframe insertion | c.8451_8452ins (NM_017780.3) | p.Asn2817dup (NP_060250.2) | rs1443281585 | | . | . | . |
| P12 | DNMT3B | 20 | het | missense | c.73G>A (NM_006892.3) | p.Gly25Arg (NP_008823.1) | rs151128145 | | 0.000762 | tolerated (0.09) | probably_damaging (0.992) |
| P12 | CFI | 4 | het | missense | c.253T>A (NM_000204.3) | p.Tyr85Asn (NP_000195.2) | . | | . | deleterious (0) | probably_damaging (0.999) |
| P12 | CSF3R | 1 | het | missense | c.2480T>C (NM_156039.3) | p.Leu827Pro (NP_724781.1) | . | | . | tolerated (0.32) | benign (0.002) |
| **Patient** | **Gene** | **Chr** | **Genotype** | **Consequence** | **cDNA** | **Protein** | **dbSNP ID** | | **ExAC freq** | **Sift** | **PolyPhen** |
| P13 | RECQL4 | 8 | het | missense | c.1954G>A (NM_004260.3) | p.Val652Met (NP_004251.3) | rs61754061 | | 0.005414 | . | probably_damaging (0.995) |
| P13 | SLC46A1 | 17 | het | missense | c.189G>C (NM_080669.4) | p.Arg63Ser (NP_542400.2) | rs41297071 | | 0.002711 | . | possibly_damaging (0.514) |
| **Patient** | **Gene** | **Chr** | **Genotype** | **Consequence** | **cDNA** | **Protein** | **dbSNP ID** | | **ExAC freq** | **Sift** | **PolyPhen** |
| P14 | LIG1 | 19 | het | missense | c.2216C>G (NM_000234.1) | p.Ser739Trp (NP_000225.1) | . | | . | deleterious (0) | probably_damaging (1) |
| P14 | USB1 | 16 | het | missense | c.74G>C (NM_024598.3) | p.Arg25Thr (NP_078874.2) | rs772792606 | | 0.000107 | tolerated (0.43) | benign (0.008) |
| **Patient** | **Gene** | **Chr** | **Genotype** | **Consequence** | **cDNA** | **Protein** | **dbSNP ID** | | **ExAC freq** | **Sift** | **PolyPhen** |
| P15 | C8A | 1 | het | missense | c.1454G>A (NM_000562.2) | p.Arg485His (NP_000553.1) | rs1620075 | | 0.00183 | deleterious (0.01) | benign (0.073) |
| P15 | ITGB2 | 21 | het | missense | c.1117G>A (NM_000211.3) | p.Ala373Thr (NP_000202.2) | rs752903416 | | 0.000025 | tolerated (0.7) | benign (0.002) |
| P15 | CD3G | 11 | het | missense | c.56G>A (NM_000073.2) | p.Gly19Asp (NP_000064.1) | rs146393315 | | 0.000634 | deleterious (0.04) | probably_damaging (0.987) |
| P15 | PRKDC | 8 | het | missense | c.3743C>T (NM_006904.6) | p.Ser1248Leu (NP_008835.5) | rs200729621 | | 0.000142 | . | . |
| **Patient** | **Gene** | **Chr** | **Genotype** | **Consequence** | **cDNA** | **Protein** | **dbSNP ID** | | **ExAC freq** | **Sift** | **PolyPhen** |
| P16 | ADA | 20 | het | missense | c.395T>C (NM_000022.2) | p.Leu132Pro (NP_000013.2) | . | | . | deleterious (0.03) | probably_damaging (0.987) |
| P16 | ITGB2 | 21 | het | missense | c.1172C>T (NM_000211.3) | p.Thr391Met (NP_000202.2) | rs141201564 | | 0.000874 | tolerated (0.09) | possibly_damaging (0.639) |
| **Patient** | **Gene** | **Chr** | **Genotype** | **Consequence** | **cDNA** | **Protein** | **dbSNP ID** | | **ExAC freq** | **Sift** | **PolyPhen** |
| P17 | ITGB2 | 21 | het | missense | c.28G>A (NM_000211.3) | p.Ala10Thr (NP_000202.2) | rs201802601 | | 0.005286 | tolerated (0.57) | benign (0.02) |
| P17 | SKIV2L | 6 | het | missense | c.3409C>T (NM_006929.4) | p.Arg1137Cys (NP_008860.4) | rs553733225 | | 0.000379 | deleterious (0.05) | probably_damaging (0.952) |
| P17 | TYK2 | 19 | het | missense | c.1534G>A (NM_003331.4) | p.Gly512Arg (NP_003322.3) | rs146786766 | | 0.000689 | tolerated (0.16) | benign (0.02) |
| **Patient** | **Gene** | **Chr** | **Genotype** | **Consequence** | **cDNA** | **Protein** | **dbSNP ID** | | **ExAC freq** | **Sift** | **PolyPhen** |
| P18 | CIITA | 16 | het | missense | c.2924A>G (NM_000246.3) | p.Lys975Arg (NP_000237.2) | rs140103491 | | 0.007423 | tolerated (0.26) | benign (0.004) |
| P18 | DOCK8 | 9 | het | missense | c.3044G>A (NM_203447.3) | p.Arg1015His (NP_982272.2) | rs200494857 | | 0.000033 | deleterious (0) | possibly_damaging (0.875) |
| P18 | TICAM1 | 19 | hom | missense | c.212G>A (NM_182919.3) | p.Arg71Gln (NP_891549.1) | rs372818181 | | 0.000504 | tolerated (0.38) | benign (0.001) |
| P18 | ZAP70 | 2 | het | missense | c.939C>G (NM_001079.3) | p.Ser313Arg (NP_001070.2) | rs145218891 | | 0.000537 | tolerated (0.44) | possibly_damaging (0.847) |
| **Patient** | **Gene** | **Chr** | **Genotype** | **Consequence** | **cDNA** | **Protein** | **dbSNP ID** | | **ExAC freq** | **Sift** | **PolyPhen** |
| P19 | C5 | 9 | het | missense | c.64A>G (NM_001735.2) | p.Thr22Ala (NP_001726.2) | rs564964646 | | 0.000774 | tolerated (0.08) | benign (0.411) |
| P19 | CIITA | 16 | het | missense | c.2384G>A (NM_000246.3) | p.Arg795Gln (NP_000237.2) | rs553503699 | | 0.000079 | tolerated (0.11) | benign (0.291) |
| P19 | TAZ | X | hom | missense | c.606G>C (NM_000116.3) | p.Glu202Asp (NP_000107.1) | rs781893396 | | 0.000023 | deleterious (0.04) | possibly_damaging (0.695) |
| **Patient** | **Gene** | **Chr** | **Genotype** | **Consequence** | **cDNA** | **Protein** | **dbSNP ID** | | **ExAC freq** | **Sift** | **PolyPhen** |
| P20 | CYBB | X | hom | missense | c.1551T>A (NM_000397.3) | p.Asp517Glu (NP_000388.2) | rs151344452 | | 0.001586 | tolerated (0.27) | benign (0.031) |
| P20 | NCF2 | 1 | het | missense | c.1184G>A (NM_000433.3) | p.Arg395Gln (NP_000424.2) | rs145229115 | | 0.001063 | tolerated (0.2) | benign (0.023) |
| **Patient** | **Gene** | **Chr** | **Genotype** | **Consequence** | **cDNA** | **Protein** | **dbSNP ID** | | **ExAC freq** | **Sift** | **PolyPhen** |
| P21 | UNC13D | 17 | het | missense | c.2341G>A (NM_199242.2) | p.Val781Ile (NP_954712.1) | rs149871493 | | 0.001269 | tolerated (0.47) | benign (0.001) |
| P21 | NFKBIA | 14 | het | missense | c.220G>C (NM_020529.2) | p.Gly74Arg (NP_065390.1) | rs766345843 | | 0.000013 | deleterious (0.02) | possibly_damaging (0.79) |
| P21 | MBL2 | 10 | het | missense | c.727G>A (NM_000242.2) | p.Val243Ile (NP_000233.1) | rs185230071 | | 0.000061 | tolerated (0.2) | benign (0.03) |
| P21 | LYST | 1 | het | missense | c.2258G>A (NM_000081.2) | p.Ser753Asn (NP_000072.2) | rs746829669 | | 0.000058 | tolerated (0.58) | benign (0.039) |
| P21 | ATM | 11 | het | missense | c.4388T>G (NM_000051.3) | p.Phe1463Cys (NP_000042.3) | rs138327406 | | 0.001378 | deleterious (0) | probably_damaging (0.946) |
| P21 | AIRE | 21 | het | missense | c.1084G>T (NM_000383.3) | p.Val362Leu (NP_000374.1) | rs763954225 | | 0.000107 | tolerated (0.48) | benign (0.047) |
| **Patient** | **Gene** | **Chr** | **Genotype** | **Consequence** | **cDNA** | **Protein** | **dbSNP ID** | | **ExAC freq** | **Sift** | **PolyPhen** |
| P22 | C5 | 9 | het | missense | c.3029C>T (NM_001735.2) | p.Ala1010Val (NP_001726.2) | rs34362143 | | 0.000659 | tolerated (0.22) | benign (0.208) |
| P22 | TICAM1 | 19 | het | missense | c.479C>T (NM_182919.3) | p.Ser160Phe (NP_891549.1) | rs145148929 | | 0.002425 | deleterious (0) | probably_damaging (0.951) |
| **Patient** | **Gene** | **Chr** | **Genotype** | **Consequence** | **cDNA** | **Protein** | **dbSNP ID** | | **ExAC freq** | **Sift** | **PolyPhen** |
| P23 | CR2 | 1 | het | missense | c.1537A>G (NM_001006658.2) | p.Ile513Val (NP_001006659.1) | rs1210123747 | | . | tolerated (0.69) | benign (0.019) |
| P23 | TERT | 5 | het | missense | c.193C>A (NM_198253.2) | p.Pro65Thr (NP_937983.2) | rs544215765 | | . | tolerated (0.4) | benign (0.255) |
| P23 | PIK3R1 | 5 | het | splice region | c.1020-3C>T (NM_181523.2) | . | rs200653607 | | 0.000266 | . | . |
| P23 | AP3B1 | 5 | het | missense | c.1069A>G (NM_003664.3) | p.Ile357Val (NP_003655.3) | rs142025324 | | 0.001484 | tolerated (0.22) | possibly_damaging (0.447) |
| P23 | PSMB8 | 6 | het | missense | c.371G>C (NM_148919.3) | p.Cys124Ser (NP_683720.2) | rs139299166 | | 0.000008 | deleterious (0) | probably_damaging (0.972) |
| P23 | CIITA | 16 | het | missense | c.326C>G (NM_000246.3) | p.Ser109Cys (NP_000237.2) | rs375019139 | | 0.000083 | deleterious (0.02) | possibly_damaging (0.705) |
| **Patient** | **Gene** | **Chr** | **Genotype** | **Consequence** | **cDNA** | **Protein** | **dbSNP ID** | | **ExAC freq** | **Sift** | **PolyPhen** |
| P24 | DNMT3B | 20 | het | missense | c.1079C>T (NM_006892.3) | p.Ser360Leu (NP_008823.1) | rs766544393 | | 0.000025 | tolerated (0.23) | benign (0.005) |
| P24 | ITCH | 20 | het | missense | c.1154C>T (NM_001257137.1) | p.Thr385Met (NP_001244066.1) | . | | . | deleterious (0) | probably_damaging (0.984) |
| **Patient** | **Gene** | **Chr** | **Genotype** | **Consequence** | **cDNA** | **Protein** | **dbSNP ID** | | **ExAC freq** | **Sift** | **PolyPhen** |
| P25 | CIITA | 16 | het | missense | c.2536C>T (NM_000246.3) | p.His846Tyr (NP_000237.2) | rs769472311 | | 0.000035 | tolerated (0.62) | benign (0.003) |
| P25 | JAK2 | 9 | het | missense | c.337C>G (NM_004972.3) | p.Leu113Val (NP_004963.1) | rs143103233 | | 0.000636 | tolerated (0.51) | benign (0.009) |
| P25 | LIG1 | 19 | het | missense | c.2535G>T (NM_000234.1) | p.Lys845Asn (NP_000225.1) | rs145821638 | | 0.000895 | deleterious (0.01) | probably_damaging (0.998) |
| **Patient** | **Gene** | **Chr** | **Genotype** | **Consequence** | **cDNA** | **Protein** | **dbSNP ID** | | **ExAC freq** | **Sift** | **PolyPhen** |
| P26 | CD8A | 2 | het | missense | c.425C>T (NM_001145873.1) | p.Ala142Val (NP_001139345.1) | rs1218870889 | | . | tolerated (0.29) | benign (0.109) |
| P26 | CFB | 6 | het | missense | c.1106C>T (NM_001710.5) | p.Pro369Leu (NP_001701.2) | rs200890358 | | 0.000491 | tolerated (0.28) | benign (0.004) |
| P26 | CIITA | 16 | het | missense | c.2924A>G (NM_000246.3) | p.Lys975Arg (NP_000237.2) | rs140103491 | | 0.007423 | tolerated (0.26) | benign (0.004) |
| P26 | CORO1A | 16 | het | missense | c.520G>A (NM_001193333.2) | p.Val174Met (NP_001180262.1) | rs769707848 | | 0.000041 | tolerated (0.49) | benign (0.022) |
| P26 | CSF3R | 1 | het | splice (donor) | c.2040+2T>C (NM_156039.3) | . | . | | . | . | . |
| P26 | ZAP70 | 2 | het | missense | c.939C>G (NM_001079.3) | p.Ser313Arg (NP_001070.2) | rs145218891 | | 0.000537 | tolerated (0.44) | possibly_damaging (0.847) |
| **Patient** | **Gene** | **Chr** | **Genotype** | **Consequence** | **cDNA** | **Protein** | **dbSNP ID** | | **ExAC freq** | **Sift** | **PolyPhen** |
| P27 | CARD9 | 9 | het | stop gained | c.1228G>T (NM_052813.4) | p.Glu410Ter (NP_434700.2) | . | | . | . | . |
| P27 | CSF3R | 1 | het | missense | c.2153C>T (NM_156039.3) | p.Pro718Leu (NP_724781.1) | rs183614500 | | 0.000158 | tolerated (0.7) | benign (0.042) |
| P27 | IL17RA | 22 | het | missense | c.152C>T (NM_014339.5) | p.Thr51Met (NP_055154.3) | rs143008696 | | 0.001738 | tolerated (0.19) | possibly_damaging (0.588) |
| P27 | IL17RA | 22 | het | missense | c.1604G>A (NM_014339.5) | p.Arg535His (NP_055154.3) | rs1415141558 | | . | tolerated (0.07) | probably_damaging (0.999) |
| P27 | LRRC8A | 9 | het | missense | c.779T>C (NM_019594.3) | p.Val260Ala (NP_062540.2) | . | | . | deleterious (0.01) | benign (0.406) |
| **Patient** | **Gene** | **Chr** | **Genotype** | **Consequence** | **cDNA** | **Protein** | **dbSNP ID** | | **ExAC freq** | **Sift** | **PolyPhen** |
| P28 | C6 | 5 | het | missense | c.1471G>A (NM_000065.2) | p.Val491Met (NP_000056.2) | rs200823179 | | 0.000198 | tolerated (0.09) | benign (0.143) |
| P28 | LIG4 | 13 | het | missense | c.1208T>C (NM_206937.1) | p.Val403Ala (NP_996820.1) | rs779305286 | | 0.000033 | tolerated (0.25) | benign (0.032) |
| P28 | CHD7 | 8 | het | missense | c.7538G>A (NM_017780.3) | p.Arg2513Gln (NP_060250.2) | rs1064794649 | | . | deleterious (0) | possibly_damaging (0.902) |
| **Patient** | **Gene** | **Chr** | **Genotype** | **Consequence** | **cDNA** | **Protein** | **dbSNP ID** | | **ExAC freq** | **Sift** | **PolyPhen** |
| P29 | C7 | 5 | het | missense | c.1852C>T (NM_000587.2) | p.Arg618Trp (NP_000578.2) | rs202140226 | | 0.000803 | deleterious (0.02) | possibly_damaging (0.549) |
| P29 | CD55 | 1 | het | missense | c.155G>C (NM_001114752.1) | p.Arg52Pro (NP_001108224.1) | rs28371588 | | 0.000272 | tolerated (0.16) | possibly_damaging (0.563) |
| P29 | KMT2D | 12 | het | missense | c.2992C>A (NM_003482.3) | p.Pro998Thr (NP_003473.3) | rs143711798 | | 0.000472 | deleterious (0) | . |
| P29 | LYST | 1 | het | missense | c.9243A>C (NM_000081.2) | p.Gln3081His (NP_000072.2) | rs757239793 | | 0.000025 | deleterious (0) | probably_damaging (0.999) |
| **Patient** | **Gene** | **Chr** | **Genotype** | **Consequence** | **cDNA** | **Protein** | | **dbSNP ID** | **ExAC freq** | **Sift** | **PolyPhen** |
| P30 | VPS13B | 8 | het | missense | c.11263C>G (NM_017890.4) | p.Leu3755Val (NP_060360.3) | rs201448515 | | 0.000021 | deleterious (0) | probably_damaging (0.992) |
| P30 | DOCK8 | 9 | het | missense | c.663C>A (NM_203447.3) | p.Asp221Glu (NP_982272.2) | rs139391329 | | 0.001731 | tolerated (1) | benign (0.001) |
| P30 | NOD2 | 16 | het | missense | c.2264C>T (NM_022162.1) | p.Ala755Val (NP_071445.1) | rs61747625 | | 0.002318 | deleterious (0.01) | probably_damaging (0.999) |
| P30 | LPIN2 | 18 | het | missense | c.2161C>T (NM_014646.2) | p.His721Tyr (NP_055461.1) | rs200256485 | | 0.000165 | deleterious (0) | probably_damaging (1) |
| **Patient** | **Gene** | **Chr** | **Genotype** | **Consequence** | **cDNA** | **Protein** | | **dbSNP ID** | **ExAC freq** | **Sift** | **PolyPhen** |
| P31 | C1R | 12 | het | missense | c.446G>A (NM_001733.4) | p.Arg149Gln (NP_001724.3) | rs73046147 | | 0.000025 | tolerated (0.95) | benign (0.001) |
| P31 | C2 | 6 | het | missense | c.386G>A (NM_000063.4) | p.Arg129His (NP_000054.2) | rs367996721 | | 0.000083 | deleterious (0) | probably_damaging (0.999) |
| P31 | CFP | X | hom | missense | c.151G>T (NM_002621.2) | p.Val51Phe (NP_002612.1) | rs751421714 | | . | deleterious (0) | possibly_damaging (0.72) |
| P31 | CIITA | 16 | het | missense | c.835A>G (NM_000246.3) | p.Thr279Ala (NP_000237.2) | rs145961289 | | 0.000132 | tolerated (0.89) | benign (0.016) |
| P31 | DOCK8 | 9 | het | missense | c.6064A>G (NM_203447.3) | p.Met2022Val (NP_982272.2) | rs143458628 | | 0.000091 | tolerated (0.17) | benign (0.03) |
| P31 | IRAK4 | 12 | het | missense | c.1169A>G (NM_016123.3) | p.His390Arg (NP_057207.2) | rs4251583 | | 0.000593 | tolerated (0.09) | benign (0.033) |
| P31 | PMM2 | 16 | het | splice (donor) | c.255+2T>C (NM_000303.2) | . | rs139716296 | | 0.000082 | . | . |
| P31 | STAT1 | 2 | het | missense | c.722G>A (NM_007315.3) | p.Arg241Gln (NP_009330.1) | rs146273341 | | 0.000222 | tolerated (0.19) | benign (0.002) |
| P31 | TICAM1 | 19 | het | missense | c.479C>T (NM_182919.3) | p.Ser160Phe (NP_891549.1) | rs145148929 | | 0.002425 | deleterious (0) | probably_damaging (0.951) |
| **Patient** | **Gene** | **Chr** | **Genotype** | **Consequence** | **cDNA** | **Protein** | | **dbSNP ID** | **ExAC freq** | **Sift** | **PolyPhen** |
| P32 | C8A | 1 | het | missense | c.89G>A (NM_000562.2) | p.Arg30Gln (NP_000553.1) | rs201955480 | | 0.000058 | tolerated (0.58) | probably_damaging (0.981) |
| P32 | DNMT3B | 20 | het | missense | c.73G>A (NM_006892.3) | p.Gly25Arg (NP_008823.1) | rs151128145 | | 0.000761 | tolerated (0.09) | probably_damaging (0.992) |
| P32 | HAX1 | 1 | het | missense | c.137G>A (NM_006118.3) | p.Arg46His (NP_006109.2) | . | | . | tolerated (0.55) | benign (0.004) |
| P32 | KMT2D | 12 | het | missense | c.1955G>C (NM_003482.3) | p.Arg652Pro (NP_003473.3) | . | | . | tolerated (0.123) | benign (0.000) |
| P32 | MPO | 17 | het | splice (acceptor) | c.2031-2A>C (NM_000250.1) | . | rs35897051 | | 0.004257 | . | . |
| P32 | TAP1 | 6 | het | stop gained | c.856C>T (NM_000593.5) | p.Arg286Ter (NP_000584.2) | . | | . | . | . |
| P32 | VPS13B | 8 | het | missense | c.2880A>T (NM_017890.4) | p.Leu960Phe (NP_060360.3) | . | | 0.000149 | tolerated (0.32) | probably_damaging (0.975) |
| **Patient** | **Gene** | **Chr** | **Genotype** | **Consequence** | **cDNA** | **Protein** | **dbSNP ID** | | **ExAC freq** | **Sift** | **PolyPhen** |
| P33 | ERCC3 | 2 | het | missense | c.144G>C (NM_000122.1) | p.Glu48Asp (NP_000113.1) | rs149309991 | | 0.000198 | tolerated (0.53) | benign (0.002) |
| P33 | PLCG2 | 16 | het | missense | c.1475C>T (NM_002661.3) | p.Thr492Ile (NP_002652.2) | rs1368273226 | | . | tolerated (0.21) | benign (0.03) |
| P33 | RAG1 | 11 | het | missense | c.101G>A (NM_000448.2) | p.Arg34Gln (NP_000439.1) | rs377307948 | | 0.000115 | deleterious (0) | possibly_damaging (0.643) |
| **Patient** | **Gene** | **Chr** | **Genotype** | **Consequence** | **cDNA** | **Protein** | **dbSNP ID** | | **ExAC freq** | **Sift** | **PolyPhen** |
| P34 | LRRC8A | 9 | het | missense | c.1250G>A (NM_019594.3) | p.Arg417Gln (NP_062540.2) | rs746556593 | | 0.000017 | tolerated (0.08) | probably_damaging (0.913) |
| P34 | CHD7 | 8 | het | missense | c.8672A>G (NM_017780.3) | p.Asn2891Ser (NP_060250.2) | rs202039728 | | 0.000216 | tolerated (0.09) | benign (0.01) |
| P34 | C4B | 6 | het | missense | c.4072C>G (NM_001002029.3) | p.Leu1358Val (NP_001002029.3) | rs747337926 | | 0.002556 | deleterious (0.01) | possibly_damaging (0.677) |
| **Patient** | **Gene** | **Chr** | **Genotype** | **Consequence** | **cDNA** | **Protein** | **dbSNP ID** | | **ExAC freq** | **Sift** | **PolyPhen** |
| P35 | IL21R | 16 | het | missense | c.681G>C (NM_181079.4) | p.Met227Ile (NP_851565.4) | rs201566028 | | 0.0002672 | . | . |
| P35 | KRAS | 12 | het | missense | c.535G>A (NM_033360.2) | p.Gly179Ser (NP_203524.1) | rs200970347 | | 0.0003626 | tolerated (0.14) | benign (0.008) |
| **Patient** | **Gene** | **Chr** | **Genotype** | **Consequence** | **cDNA** | **Protein** | **dbSNP ID** | | **ExAC freq** | **Sift** | **PolyPhen** |
| P37 | CD19 | 16 | het | missense | c.395T>G (NM_001178098.1) | p.Leu132Arg (NP_001171569.1) | rs146795664 | | 0.002125 | tolerated (0.63) | benign (0.015) |
| P37 | CTLA4 | 2 | het | missense | c.326G>A (NM_005214.4) | p.Gly109Glu (NP_005205.2) | rs144988077 | | 0.000239 | tolerated (0.56) | benign (0.05) |
| P37 | LIG4 | 13 | het | missense | c.2425C>G (NM_206937.1) | p.Pro809Ala (NP_996820.1) | rs137899041 | | 0.000082 | tolerated (0.19) | benign (0.003) |
| P37 | TICAM1 | 19 | het | missense | c.238G>A (NM_182919.3) | p.Val80Met (NP_891549.1) | rs199816697 | | 0.000714 | tolerated (0.35) | benign (0.005) |
| **Patient** | **Gene** | **Chr** | **Genotype** | **Consequence** | **cDNA** | **Protein** | **dbSNP ID** | | **ExAC freq** | **Sift** | **PolyPhen** |
| P38 | PSTPIP1 | 15 | het | missense | c.525C>G (NM_003978.3) | p.Asn175Lys (NP_003969.2) | . | | . | tolerated (0.48) | benign (0.049) |
| P38 | VPS13B | 8 | het | missense | c.5957G>A (NM_017890.4) | p.Gly1986Glu (NP_060360.3) | rs1440579372 | | . | deleterious (0) | probably_damaging (0.997) |
| **Patient** | **Gene** | **Chr** | **Genotype** | **Consequence** | **cDNA** | **Protein** | **dbSNP ID** | | **ExAC freq** | **Sift** | **PolyPhen** |
| P39 | TICAM1 | 19 | het | missense | c.479C>T (NM_182919.3) | p.Ser160Phe (NP_891549.1) | rs145148929 | | 0.002425 | deleterious (0) | probably_damaging (0.951) |
| **Patient** | **Gene** | **Chr** | **Genotype** | **Consequence** | **cDNA** | **Protein** | **dbSNP ID** | | **ExAC freq** | **Sift** | **PolyPhen** |
| P40 | KMT2D | 12 | het | missense | c.14438A>G (NM_003482.3) | p.Asn4813Ser (NP_003473.3) | rs756696295 | | 0.000008 | tolerated (0.39) | benign (0.004) |
| P40 | ALG13 | X | het | missense | c.3249C>A (NM_001099922.2) | p.Asp1083Glu (NP_001093392.1) | . | | . | deleterious (0.04) | probably_damaging (0.946) |
| **Patient** | **Gene** | **Chr** | **Genotype** | **Consequence** | **cDNA** | **Protein** | **dbSNP ID** | | **ExAC freq** | **Sift** | **PolyPhen** |
| P41 | FOXN1 | 17 | het | missense | c.8C>T (NM_003593.2) | p.Ser3Leu (NP_003584.2) | rs146091703 | | 0.000026 | deleterious (0) | probably_damaging (0.975) |
| P41 | C2 | 6 | het | missense | c.614G>A (NM_000063.4) | p.Arg205His (NP_000054.2) | rs147186833 | | 0.000202 | deleterious (0.01) | probably_damaging (0.959) |
| P41 | ITGB2 | 21 | het | missense | c.1172C>T (NM_000211.3) | p.Thr391Met (NP_000202.2) | rs141201564 | | 0.000874 | tolerated (0.09) | possibly_damaging (0.639) |
| P41 | CTSC | 11 | het | missense | c.1201G>A (NM_001814.4) | p.Glu401Lys (NP_001805.3) | rs200627023 | | 0.000107 | tolerated (0.09) | possibly_damaging (0.452) |
| P41 | TCIRG1 | 11 | het | missense | c.1615G>A (NM_006019.3) | p.Val539Ile (NP_006010.2) | rs142539969 | | 0.000157 | tolerated (0.5) | benign (0.099) |
| P41 | UNC13D | 17 | het | missense | c.2341G>A (NM_199242.2) | p.Val781Ile (NP_954712.1) | rs149871493 | | 0.001269 | tolerated (0.47) | benign (0.001) |
| P41 | PRKDC | 8 | het | missense | c.1338C>A (NM_006904.6) | p.Phe446Leu (NP_008835.5) | rs61729514 | | 0.000879 | . | probably_damaging (0.999) |
| **Patient** | **Gene** | **Chr** | **Genotype** | **Consequence** | **cDNA** | **Protein** | **dbSNP ID** | | **ExAC freq** | **Sift** | **PolyPhen** |
| P42 | CEBPE | 14 | het | missense | c.410G>A (NM_001805.3) | p.Ser137Asn (NP_001796.2) | rs140606768 | | 0.000346 | tolerated (0.52) | benign (0.001) |
| P42 | VPS13B | 8 | het | missense | c.4774G>A (NM_017890.4) | p.Ala1592Thr (NP_060360.3) | . | | . | deleterious (0.03) | probably_damaging (0.966) |
| P42 | C7 | 5 | het | missense | c.659G>A (NM_000587.2) | p.Arg220Gln (NP_000578.2) | rs369349760 | | 0.000167 | tolerated (0.58) | benign (0.024) |
| P42 | LYST | 1 | het | missense | c.8214G>C (NM_000081.2) | p.Glu2738Asp (NP_000072.2) | rs140944484 | | 0.000207 | tolerated (1) | benign (0.002) |
| **Patient** | **Gene** | **Chr** | **Genotype** | **Consequence** | **cDNA** | **Protein** | **dbSNP ID** | | **ExAC freq** | **Sift** | **PolyPhen** |
| P43 | KMT2D | 12 | het | missense | c.1967T>C (NM_003482.3) | p.Leu656Pro (NP_003473.3) | rs1169567206 | | . | tolerated (0.06) | benign (0) |
| **Patient** | **Gene** | **Chr** | **Genotype** | **Consequence** | **cDNA** | **Protein** | **dbSNP ID** | | **ExAC freq** | **Sift** | **PolyPhen** |
| P44 | LYST | 1 | het | missense | c.5676G>A (NM_000081.2) | p.Met1892Ile (NP_000072.2) | rs143857674 | | 0.000041 | tolerated (0.98) | benign (0) |
| P44 | TMC8 | 17 | het | missense | c.1168G>A (NM_152468.4) | p.Val390Ile (NP_689681.2) | rs150546646 | | 0.001003 | tolerated (0.1) | benign (0.009) |
| P44 | DNMT3B | 20 | het | missense | c.1345C>G (NM_006892.3) | p.Leu449Val (NP_008823.1) | rs757117952 | | 0.000008 | deleterious (0) | probably_damaging (0.964) |
| P44 | DNMT3B | 20 | het | missense | c.1352A>T (NM_006892.3) | p.Glu451Val (NP_008823.1) | . | | . | tolerated (0.19) | benign (0.02) |
| **Patient** | **Gene** | **Chr** | **Genotype** | **Consequence** | **cDNA** | **Protein** | **dbSNP ID** | | **ExAC freq** | **Sift** | **PolyPhen** |
| P45 | ZBTB24 | 6 | het | missense | c.855G>T (NM_014797.2) | p.Arg285Ser (NP_055612.2) | rs143216162 | | 0.0000494 | deleterious (0.03) | benign (0.039) |
| P45 | NCF1 | 7 | het | missense | c.292T>G (NM_000265.4) | p.Cys98Gly (NP_000256.3) | rs144018361 | | 0.0007298 | deleterious (0.02) | possibly_damaging (0.849) |
| P45 | CHD7 | 8 | het | missense | c.5848G>A (NM_017780.3) | p.Ala1950Thr (NP_060250.2) | rs201423234 | | 0.0003115 | tolerated (0.64) | benign (0.032) |
| P45 | TNFRSF1A | 12 | het | missense | c.1354A>G (NM_001065.3) | p.Ser452Gly (NP_001056.1) | rs200346150 | | 0.000012 | tolerated (0.14) | benign (0) |
| P45 | AIRE | 21 | het | missense | c.1249C>A (NM_000383.3) | p.Leu417Ile (NP_000374.1) | . | | . | tolerated (0.06) | probably_damaging (0.971) |
| **Patient** | **Gene** | **Chr** | **Genotype** | **Consequence** | **cDNA** | **Protein** | **dbSNP ID** | | **ExAC freq** | **Sift** | **PolyPhen** |
| P46 | FOXN1 | 17 | het | missense | c.1727C>T (NM_003593.2) | p.Pro576Leu (NP_003584.2) | rs756165992 | | 0.000025 | tolerated (0.15) | probably_damaging (0.987) |
| P46 | PEPD | 19 | het | missense | c.794G>A (NM_000285.3) | p.Arg265Gln (NP_000276.2) | rs200351927 | | 0.000203 | tolerated (0.11) | benign (0.148) |
| P46 | TAP1 | 6 | het | missense | c.919G>A (NM_000593.5) | p.Gly307Arg (NP_000584.2) | rs59328013 | | 0.000121 | deleterious (0.03) | probably_damaging (0.949) |
| P46 | SKIV2L | 6 | het | missense | c.2564C>A (NM_006929.4) | p.Ala855Glu (NP_008860.4) | rs561701823 | | 0.000034 | tolerated (0.48) | benign (0.076) |
| P46 | CTSC | 11 | het | missense | c.113C>G (NM_001814.4) | p.Thr38Ser (NP_001805.3) | rs758568173 | | 0.000075 | tolerated (0.31) | benign (0.057) |
| P46 | TBK1 | 12 | het | missense | c.1522C>A (NM_013254.3) | p.Leu508Ile (NP_037386.1) | rs144424516 | | 0.000776 | tolerated (0.46) | benign (0.033) |
| P46 | STK4 | 20 | het | missense | c.47A>G (NM_006282.2) | p.Lys16Arg (NP_006273.1) | rs142594802 | | 0.000206 | tolerated (0.11) | benign (0.402) |
| **Patient** | **Gene** | **Chr** | **Genotype** | **Consequence** | **cDNA** | **Protein** | **dbSNP ID** | | **ExAC freq** | **Sift** | **PolyPhen** |
| P47 | CHD7 | 8 | het | missense | c.602A>G (NM_017780.3) | p.Gln201Arg (NP_060250.2) | rs764496155 | | 0.000092 | tolerated (0.27) | benign (0) |
| P47 | UNC13D | 17 | het | missense | c.869C>T (NM_199242.2) | p.Ser290Leu (NP_954712.1) | rs202020396 | | 0.000079 | tolerated (0.22) | benign (0.01) |
| P47 | VPS13B | 8 | het | missense | c.1559A>G (NM_017890.4) | p.His520Arg (NP_060360.3) | rs143205296 | | 0.000538 | deleterious (0) | possibly_damaging (0.897) |
| **Patient** | **Gene** | **Chr** | **Genotype** | **Consequence** | **cDNA** | **Protein** | **dbSNP ID** | | **ExAC freq** | **Sift** | **PolyPhen** |
| P48 | PLCG2 | 16 | het | missense | c.1258G>A (NM_002661.3) | p.Ala420Thr (NP_002652.2) | rs201490178 | | 0.00431 | tolerated (0.39) | benign (0.004) |
| P48 | C5 | 9 | het | missense | c.3463G>A (NM_001735.2) | p.Ala1155Thr (NP_001726.2) | rs200624729 | | 0.000382 | deleterious (0.01) | possibly_damaging (0.844) |
| P48 | C6 | 5 | het | missense | c.1355G>A (NM_000065.2) | p.Gly452Glu (NP_000056.2) | rs142896559 | | 0.004985 | deleterious (0.01) | benign (0.003) |
| P48 | ZAP70 | 2 | het | missense | c.939C>G (NM_001079.3) | p.Ser313Arg (NP_001070.2) | rs145218891 | | 0.000537 | tolerated (0.44) | possibly_damaging (0.847) |
| P48 | KMT2D | 12 | het | missense | c.6844C>T (NM_003482.3) | p.Arg2282Trp (NP_003473.3) | rs587783726 | | 0.000042 |  | possibly_damaging |
| **Patient** | **Gene** | **Chr** | **Genotype** | **Consequence** | **cDNA** | **Protein** | **dbSNP ID** | | **ExAC freq** | **Sift** | **PolyPhen** |
| P49 | UNC13D | 17 | het | missense | c.2341G>A (NM_199242.2) | p.Val781Ile (NP_954712.1) | rs149871493 | | 0.001269 | tolerated (0.47) | benign (0.001) |
| P49 | CFH | 1 | het | missense | c.1548T>A (NM_000186.3) | p.Asn516Lys (NP_000177.2) | rs147403664 | | 0.000405 | tolerated (0.11) | probably_damaging (0.977) |
| P49 | TAP1 | 6 | het | splice region | c.1231-3C>T (NM_000593.5) | . | rs56366814 | | 0.003445 | . | . |
| P49 | RAG2 | 11 | het | missense | c.22G>A (NM_000536.3) | p.Val8Ile (NP_000527.2) | rs150762709 | | 0.003189 | tolerated (0.41) | possibly_damaging (0.842) |
| **Patient** | **Gene** | **Chr** | **Genotype** | **Consequence** | **cDNA** | **Protein** | **dbSNP ID** | | **ExAC freq** | **Sift** | **PolyPhen** |
| P50 | SKIV2L | 6 | het | missense | c.797C>G (NM_000063.4) | p.Ser266Trp (NP_000054.2) | rs116568722 | | 0.000214 | deleterious (0) | probably_damaging (0.962) |
| P50 | C3 | 19 | het | missense | c.2203C>T (NM_000064.2) | p.Arg735Trp (NP_000055.2) | rs117793540 | | 0.002086 | deleterious (0) | probably_damaging (0.99) |
| **Patient** | **Gene** | **Chr** | **Genotype** | **Consequence** | **cDNA** | **Protein** | **dbSNP ID** | | **ExAC freq** | **Sift** | **PolyPhen** |
| P51 | KMT2D | 12 | het | missense | c.7670C>T (NM_003482.3) | p.Pro2557Leu (NP_003473.3) | rs189888707 | | 0.008494 |  | probably damaging (0.998) |
| P51 | TCIRG1 | 11 | het | missense | c.479G>A (NM_006019.3) | p.Gly160Glu (NP_006010.2) | rs186758849 | | 0.005491 | tolerated (0.61) | benign (0.076) |
| **Patient** | **Gene** | **Chr** | **Genotype** | **Consequence** | **cDNA** | **Protein** | **dbSNP ID** | | **ExAC freq** | **Sift** | **PolyPhen** |
| P52 | TNFRSF11A | 18 | het | missense | c.1279G>A (NM_003839.2) | p.Asp427Asn (NP_003830.1) | rs201402594 | | 0.000796 | tolerated (0.06) | benign (0.238) |
| P52 | LPIN2 | 18 | het | missense | c.1876C>T (NM_014646.2) | p.Pro626Ser (NP_055461.1) | rs150806357 | | 0.002973 | tolerated (0.69) | benign (0.003) |
| **Patient** | **Gene** | **Chr** | **Genotype** | **Consequence** | **cDNA** | **Protein** | **dbSNP ID** | | **ExAC freq** | **Sift** | **PolyPhen** |
| P53 | C1QA | 1 | het | missense | c.295A>C (NM_015991.2) | p.Ile99Leu (NP_057075.1) | rs180679721 | | 0.000304 | tolerated (0.35) | benign (0.001) |
| P53 | PTPRC | 1 | het | missense | c.260C>T (NM_002838.4) | p.Pro87Leu (NP_002829.3) | rs149798940 | | 0.000074 | tolerated (0.71) | benign (0) |
| P53 | TERT | 5 | het | missense | c.1234C>T (NM_198253.2) | p.His412Tyr (NP_937983.2) | rs34094720 | | 0.006342 | tolerated (0.21) | possibly_damaging (0.885) |
| P53 | SKIV2L | 6 | het | missense | c.1705G>A (NM_006929.4) | p.Val569Met (NP_008860.4) | rs144147284 | | 0.002596 | tolerated (0.07) | possibly_damaging (0.581) |
| P53 | RFXANK | 19 | het | missense | c.187G>A (NM_003721.2) | p.Ala63Thr (NP_003712.1) | rs150525759 | | 0.000074 | tolerated (0.58) | benign (0.001) |
| **Patient** | **Gene** | **Chr** | **Genotype** | **Consequence** | **cDNA** | **Protein** | **dbSNP ID** | | **ExAC freq** | **Sift** | **PolyPhen** |
| P54 | TRAF3 | 14 | het | missense | c.74G>T (NM_003300.3) | p.Arg25Leu (NP_003291.2) | rs370955205 | | 0.000189 | tolerated (0.17) | possibly_damaging (0.822) |
| P54 | NOD2 | 16 | het | missense | c.1627C>T (NM_022162.1) | p.Arg543Cys (NP_071445.1) | rs545580252 | | 0.000041 | tolerated (0.09) | benign (0.008) |
| P54 | KMT2D | 12 | het | missense | c.10469C>T (NM_003482.3) | p.Pro3490Leu (NP_003473.3) | rs376980951 | | 0.000126 | deleterious (0.01) | possibly_damaging (0.483) |
| P54 | ITK | 5 | het | missense | c.1510A>T (NM_005546.3) | p.Thr504Ser (NP_005537.3) | rs151046132 | | 0.000824 | tolerated (0.48) | benign (0.037) |
| P54 | MTHFD1 | 14 | het | missense | c.920A>G (NM_005956.3) | p.Tyr307Cys (NP_005947.3) | rs577555809 | | 0.001188 | tolerated (0.19) | possibly_damaging (0.871) |
| P54 | PRKDC | 8 | het | missense | c.9445G>A (NM_006904.6) | p.Ala3149Thr (NP_008835.5) | rs8178208 | | 0.008821 | . | . |
| P54 | ATM | 11 | het | missense | c.1274C>A (NM_000051.3) | p.Ala425Glu (NP_000042.3) | . | | . | tolerated (1) | benign (0.001) |
| **Patient** | **Gene** | **Chr** | **Genotype** | **Consequence** | **cDNA** | **Protein** | **dbSNP ID** | | **ExAC freq** | **Sift** | **PolyPhen** |
| P55 | FAS | 10 | het | missense | c.580G>A (NM_000043.4) | p.Glu194Lys (NP_000034.1) | rs56006128 | | 0.001801 | tolerated (0.95) | benign (0) |
| P55 | DOCK8 | 9 | het | missense | c.287C>T (NM_203447.3) | p.Thr96Met (NP_982272.2) | . | | . | tolerated (0.1) | possibly_damaging (0.793) |
| **Patient** | **Gene** | **Chr** | **Genotype** | **Consequence** | **cDNA** | **Protein** | **dbSNP ID** | | **ExAC freq** | **Sift** | **PolyPhen** |
| P56 | IFNGR2 | 21 | het | missense | c.439G>A (NM_005534.3) | p.Glu147Lys (NP_005525.2) | rs17878639 | | 0.00152 | tolerated (0.93) | benign (0.156) |
| **Patient** | **Gene** | **Chr** | **Genotype** | **Consequence** | **cDNA** | **Protein** | **dbSNP ID** | | **ExAC freq** | **Sift** | **PolyPhen** |
| P57 | C8A | 1 | het | missense | c.385G>A (NM_000562.2) | p.Asp129Asn (NP_000553.1) | rs150404785 | | 0.00223 | tolerated (0.39) | benign (0.059) |
| **Patient** | **Gene** | **Chr** | **Genotype** | **Consequence** | **cDNA** | **Protein** | **dbSNP ID** | | **ExAC freq** | **Sift** | **PolyPhen** |
| P58 | C1S | 12 | het | missense | c.943G>A (NM_001734.3) | p.Asp315Asn (NP_001725.1) | rs117907409 | | 0.003419 | deleterious (0) | probably damaging (1) |
| P58 | DNMT3B | 20 | het | missense | c.1150G>A (NM_006892.3) | p.Ala384Thr (NP_008823.1) | rs150682895 | | 0.007936 | tolerated (1) | benign (0.007) |
| P58 | KMT2D | 12 | het | missense | c.13258C>T (NM_003482.3) | p.Arg4420Trp (NP_003473.3) | rs199797812 | | 0.000059 | deleterious (0.00) | probably damaging (1) |
| P58 | CFH | 1 | het | missense | c.2850G>T (NM_000186.3) | p.Gln950His (NP_000177.2) | rs149474608 | | 0.003583 | deleterious (0.04) | probably damaging (0.962) |
| P58 | STX11 | 6 | het | missense | c.799G>A (NM_003764.3) | p.Val267Met (NP_003755.2) | rs45574234 | | 0.00562 | tolerated (0.08) | possibly damaging (0.847) |
| P58 | PRKDC | 8 | het | missense | c.6479C>T (NM_006904.6) | p.Thr2160Met (NP_008835.5) | rs55991828 | | 0.004512 | . | . |
| P58 | C7 | 5 | het | missense | c.2008G>A (NM_000587.2) | p.Ala670Thr (NP_000578.2) | rs200737768 | | 0.000725 | tolerated (0.79) | benign (0.004) |
| P58 | C9 | 5 | het | missense | c.499C>T (NM_001737.3) | p.Pro167Ser (NP_001728.1) | rs34882957 | | 0.004711 | deleterious (0) | probably damaging (0.971) |
| **Patient** | **Gene** | **Chr** | **Genotype** | **Consequence** | **cDNA** | **Protein** | **dbSNP ID** | | **ExAC freq** | **Sift** | **PolyPhen** |
| P59 | STAT1 | 2 | het | missense | c.722G>A (NM_007315.3) | p.Arg241Gln (NP_009330.1) | rs146273341 | | 0.000222 | tolerated (0.18) | benign (0.182) |
| P59 | PRKDC | 8 | het | missense | c.16G>T (NM_006904.6) | p.Ala6Ser (NP_008835.5) | rs8177999 | | 0.006118 | . | benign (0.003) |
| P59 | JAK2 | 9 | het | missense | c.1711G>A (NM_004972.3) | p.Gly571Ser (NP_004963.1) | rs139504737 | | 0.000481 | tolerated (0.15) | probably damaging (0.974) |
| P59 | PLCG2 | 16 | het | missense | c.2011A>G (NM_002661.3) | p.Ile671Val (NP_002652.2) | rs150833842 | | 0.006124 | tolerated (1) | benign (0.016) |
| P59 | CIITA | 16 | het | missense | c.3317C>T (NM_000246.3) | p.Ala1106Val (NP_000237.2) | rs1231888573 | | . | tolerated (0.17) | possibly damaging (0.611) |
| **Patient** | **Gene** | **Chr** | **Genotype** | **Consequence** | **cDNA** | **Protein** | **dbSNP ID** | | **ExAC freq** | **Sift** | **PolyPhen** |
| P60 | PSTPIP1 | 15 | het | missense | c.657A>C (NM_003978.3) | p.Gln219His (NP_003969.2) | rs139362350 | | 0.000702 | deleterious (0) | probably damaging (0.94) |
| P60 | CHD7 | 8 | het | missense | c.8740G>A (NM_017780.3) | p.Gly2914Arg (NP_060250.2) | rs187751757 | | 0.000352 | . | probably damaging (0.998) |
| P60 | VPS13B | 8 | het | missense | c.5350A>T (NM_017890.4) | p.Thr1784Ser (NP_060360.3) | . | | . | tolerated (0.61) | possibly damaging (0.899) |
| **Patient** | **Gene** | **Chr** | **Genotype** | **Consequence** | **cDNA** | **Protein** | **dbSNP ID** | | **ExAC freq** | **Sift** | **PolyPhen** |
| P61 | PLCG2 | 16 | het | missense | c.3786G>C (NM_002661.3) | p.Lys1262Asn (NP_002652.2) | rs374430619 | | 0.000008 | deleterious (0.01) | benign (0.045) |
| P61 | NLRP12 | 19 | het | missense | c.857C>T (NM_144687.2) | p.Pro286Leu (NP_653288.1) | rs201940393 | | 0.000099 | deleterious (0) | probably_damaging (0.971) |
| P61 | GFI1 | 1 | het | missense | c.319C>G (NM_001127216.1) | p.Pro107Ala (NP_001120688.1) | rs149914857 | | 0.002583 | deleterious (0.01) | benign (0.239) |
| P61 | G6PC3 | 17 | het | missense | c.403A>G (NM_138387.3) | p.Thr135Ala (NP_612396.1) | . | | . | tolerated (0.77) | benign (0.001) |

Predicted loss-of-function variants are in blue.
